# Have bird distributions shifted along an elevational gradient on a tropical mountain?

**DOI:** 10.1002/ece3.3520

**Published:** 2017-10-20

**Authors:** Marconi Campos‐Cerqueira, Wayne J. Arendt, Joseph M. Wunderle, T. Mitchell Aide

**Affiliations:** ^1^ University of Puerto Rico‐Río Piedras San Juan Puerto Rico; ^2^ International Institute of Tropical Forestry USDA Forest Service Sabana Field Research Station Luquillo Puerto Rico

**Keywords:** acoustic monitoring, ARBIMON, climate change, occupancy

## Abstract

An upward shift in elevation is one of the most conspicuous species responses to climate change. Nevertheless, downward shifts and, apparently, the absences of response have also been recently reported. Given the growing evidence of multiple responses of species distributions due to climate change and the paucity of studies in the tropics, we evaluated the response of a montane bird community to climate change, without the confounding effects of land‐use change. To test for elevational shifts, we compared the distribution of 21 avian species in 1998 and 2015 using occupancy models. The historical data set was based on point counts, whereas the contemporary data set was based on acoustic monitoring. We detected a similar number of species in historical (36) and contemporary data sets (33). We show an overall pattern of no significant change in range limits for most species, although there was a significant shift in the range limit of eight species (38%). Elevation limits shifted mostly upward, and this pattern was more common for upper than lower limits. Our results highlight the variability of species responses to climate change and illustrate how acoustic monitoring provides an easy and powerful way to monitor animal populations along elevational gradients.

## 1 INTRODUCTION

There is now ample evidence that climate change is altering species distributions throughout the globe (Chen, Hill, Ohlemüller, Roy, & Thomas, 2011; Hughes, 2000; Lenoir, Gégout, Marquet, de Ruffray, & Brisse, 2008; Parmesan, 2006; Parmesan & Yohe, 2003; Walther et al., 2002). In montane environments, the most commonly documented spatial pattern associated with climate change is a general upward shift in species distribution. Species are shifting to higher elevation areas at a median rate of 11 meters per decade (Chen, Hill, Ohlemüller, et al., 2011), and although upward shifts have been observed both in temperate and tropical mountains, much less documentation exists for the tropics (Lenoir & Svenning, 2014). The paucity of studies in the tropics may compromise our predictions of species extinctions risk (Maclean & Wilson, 2011) and undermine our ability to manage these ecosystems. As climate change is expected to push species up the mountains, and given that tropical species are already showing higher levels of local extinctions due to climate changes (Wiens, 2016), there is an urgent need to monitor species responses through the entire elevational gradient, especially in the tropics, to support conservation planning.

Even though many montane species have already shifted their distribution upward, there are also an increasing number of studies documenting different patterns of changes in species distributions. For instance, spatially heterogeneous shifts of birds and small mammals were detected in Sierra Nevada Mountains, USA (Rowe et al., 2015; Tingley, Koo, Moritz, Rush, & Beissinger, 2012) demonstrating that individual species respond differently across geographically replicated landscapes. Downward shifts in elevation were also observed in the Appalachian Mountains where 9 of 11 high‐elevation species have shifted their lower elevational range downward, probably following changes in vegetation composition (Deluca & King, 2016). A downward shift in optimum elevation was also observed for plants species in California, where species were tracking water availability (Crimmins, Dobrowski, Greenberg, Abatzoglou, & Mynsberge, 2011). In the French Alps, a 2.3°C increase in spring temperatures did not result in a significant upward shift in the distribution of the bird community (Archaux, 2004). These diverse responses reflect a more complex phenomenon, where many other factors, such as biotic interactions (Hättenschwiler & Körner, 1995; Merrill et al., 2008; Van der Putten, Macel, & Visser, 2010), stochastic population fluctuations (Lenoir et al., 2010), and land‐use change (Archaux, 2004; Jetz, Wilcove, & Dobson, 2007), affect species elevational distribution.

Given the high conservation priority of tropical montane ecosystems, and the growing evidence of multiple responses in species distributions, we compared past and contemporary elevational distributions of birds in the Luquillo Mountains, Puerto Rico, to test for shifts in bird distributions. We predicted that species would shift their elevational distribution upward relative to their historical elevational distribution given that the average temperature in Puerto Rico has increased by approximately 2.24°C from 1950 to 2014 (Méndez‐Tejeda, 2017).

To test our hypothesis, we compared the elevational distribution of 21 species in 1998 and in 2015. The historical data set was based on point counts, whereas the contemporary data set was based on acoustic monitoring. Occupancy models were used to take into account differences related with the sampling process between the two time periods.

## 2 METHODS

### 2.1 Study site

**Figure 1:**
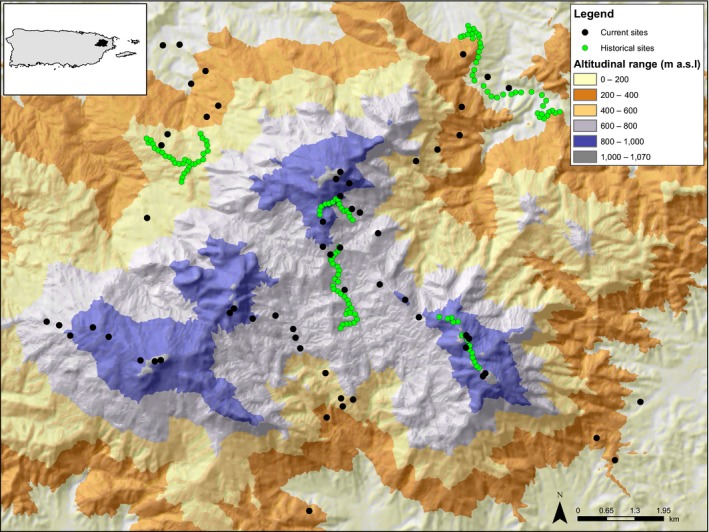
Map of the Luquillo Mountains and its location in NE Puerto Rico (black area in the inserted map). The black circles represent sites sampled in 2015 and yellow circles sites with historical data. Different colors represent differences in elevation (m a.s.l.)

The study was conducted in the Luquillo Mountains (LM) in north‐eastern Puerto Rico (Figure 1). Most of the LM are protected by the El Yunque National Forest, also known as the Luquillo Experimental Forest, which is the largest protected area (115 km^2^) with primary forest in Puerto Rico (Lugo,^1994^) and spans an elevational range from 100 to 1,074 m. This protected site is ideal for testing for elevational shifts for three reasons: (1) there are no direct effects of land‐use change during the last 100 years in the LM; (2) the LM comprises three main peaks (Pico del Este—1,051 m, Pico del Yunque—1,050 m, Pico del Toro—1,074 m) providing several elevational gradients; (3) there is extensive research documenting abiotic and biotic changes along these elevational gradients. For instance, the LM elevational gradient has a positive relationship with rainfall, runoff, humidity, cloud cover, and wind velocity (Briscoe, 1966; García‐Martinó, Warner, Scatena, & Civco, 1996; Weaver & Gould, 2013) and a negative relationship with temperature, forest growth, and canopy height (Wang, Hall, Scatena, Fetcher, & Wu, 2003; Weaver, 2000; Weaver & Gould, 2013; Weaver & Murphy, 1990). Average temperature declines with elevation from approximately 26.5°C in the lowlands to approximately 20°C at the summit (Waide, Comarazamy, González, Hall, & Lugo, 2013). Average annual rainfall ranges from 2,450 mm/year at lower elevations to over 4,000 mm/year at higher elevation sites (Waide et al., 2013). In addition, the distribution of plants and animals is also strongly affected by this elevation gradient (Brokaw et al., 2012; Campos‐Cerqueira & Aide, 2016; González et al., 2007; Gould, Gonzalez, & Rivera, 2006; Gould et al., 2008; Weaver & Gould, 2013; Willig et al., 2011).

### 2.2 Climate change in Puerto Rico and the Luquillo Mountains

The average temperature in Puerto Rico has increased by approximately 2.24°C from 1950 to 2014, with an increase in minimum temperature of 0.048°C/year and an increase in maximum temperature 0.022°C/year (Méndez‐Tejeda, 2017). Studies conducted at the local and regional scale encompassing the LM have shown a significant increase in annual mean temperature (0.007°C/year) during the 62 year period between 1932 and 1994 in the lowlands (100–450 m) of the LM (Greenland & Kittel, 2002); an increasing trend in the minimum daily temperature at El Verde Station (350–450 m) of 0.02°C/year from 1970 to 2000 (Burrowes, Joglar, & Green, 2004); and a significant increase in the mean minimum temperature between 1970 and 2000 on the East Peak summit (1,051 m) (Lasso & Ackerman, 2003). In addition, a short‐term study from 2001 to 2013 along the elevational gradient in El Yunque showed an increase in the daily minimum temperature (~0.02°C/year) across the elevation gradient (Van Beusekom, González, & Rivera, 2015). Together, these independent sources provide strong evidence for an increase in temperature in our study area.

### 2.3 Survey and resurvey data

The historical distribution data were based on the long‐term forest bird population study (1989–2006) led by WJA (Arendt, Qian, & Mineard, 2013). The sampling sites (*n* = 105) were spaced at least 100 m apart and were distributed along an elevational gradient (151–1,011 m) in the LM. At each site, birds were sampled visually and aurally for 10 min in fixed‐radii concentric circles between 05:00 and 10:30 hr. To eliminate double counting of vociferous species such as parrots and thrashers, we truncated data to detections with distances ≤40 m and grouped observations by 0–10, 11–20, and 21–40. Surveyors recorded the number of individuals of all species detected during the count, estimated distance to detected birds or to the center of the cluster, that is, groups of two or more (Thomas et al., 2010). Aerial birds were not recorded unless they perched in vegetation during the 10‐min count. Surveys were conducted approximately monthly from 1989 to 2006. To minimize observer bias, three field biologists conducted most of the surveys over the 17‐year period.

Although the historical data include 17 years of bird monitoring, for this study, we used the data from February to June 1998 (with at least three surveys for each sampling site), for comparison with the 2015 acoustic survey. The main reasons for using the 1998 data were: (1) This sampling period occurred 10 years after Hurricane Hugo, and a few months before Hurricane Georges, thus reducing any extreme variation in the elevational distributions due to the hurricanes. (2) About 81% of the detections from this year were made by only one observer, minimizing observer bias. (3) The time period, February to June, of the 1998 survey closely coincided with the March to May 2015 acoustic census. In this way, it is easier to assume population closure within each time period (an essential assumption in occupancy models), as most birds breed from March to June (Reagan & Waide, 1996).

The contemporary distribution data derive from 60 sampling sites in the LM along three elevational transects (85–1,047 m, ~20 sampling sites per elevational transect) between March and May of 2015. There were no cyclonic storms (i.e., tropical storms, hurricanes) during this sampling period. The elevational transects took advantage of roads and trails, but recorders were placed more than 200 m from any road. Each elevational transect started in the lowlands and reached one of the three main mountain peaks of the LM (Pico del Este, Pico del Yunque and Pico del Toro). Along each elevational transect, two recorders, separated by at least 200 m, were deployed at 100‐m elevation intervals. In this way, all sampling sites from each transect (~20 sampling sites per elevational transect) were sampled simultaneously using 20 audio recorders. Recorders collected data at each site for approximately 1 week and were then moved to another elevation transect.

ARBIMON portable recorders (LG smartphone enclosed in a waterproof case with an external connector linked to a Monoprice microphone) running the ARBIMON Touch application (https://play.google.com/store) were used to collect the audio recording. Recorders were placed on trees at the height of 1.5 m and programed to record 1 min of audio every 10 min for a total of 144, 1‐min recordings per day. The recording system used a sampling rate of 44.1 kHz/16 bit, which recorded sounds between 0 and 22 kHz, the frequency range of most bird species. To estimate the detection area within the study area, we played bird songs at different distances from the recorder, and we have found that most bird species were detected up to approximately 50 m. Therefore, a site is defined as a three‐dimensional hemisphere with a radius of approximately 50 m around the recorder. This sampling area is similar to the sampling area from the point counts, as the outer band of point counts terminated at a distance of 40 m. All recordings were analyzed, permanently stored, and are available in the ARBIMON project (arbimon.sieve‐analytics.com/project/elevation/dashboard).

The detection of the species in the audio recordings followed two steps: (1) First author (MCC) listened to all recordings from 05:30 to 10:00 hr, and then, one recording every hour (e.g., 11:00, 12:00, 13:00 hr) until 18:00 hr on the first survey day for each sampling site (*n* = 35, 1‐min recordings/site) for a total of 2,100 validated recordings, and (2) with a preliminary bird list from the first survey day from each site, MCC visually scanned all spectrograms of recordings (Campos‐Cerqueira & Aide, 2016; Truskinger, Cottman‐Fields, Johnson, & Roe, 2013), from 05:30 to 18:00 hr for all subsequent days, thus an additional approximately 41,000 1 min recordings were evaluated. During this process, MCC listened to every recording that included a previously unidentified vocalization for the site. Vocalizations that could not be identified by MCC were reviewed by the other authors.

### 2.4 Species data set

Although the number of species detected in both time periods was similar (37 historical species, 33 contemporary species), we focused our comparisons on species that had at least two detections in each sampling periods. This resulted in the analyses of the elevational distributions of 21 species. The Red‐tailed hawk (*Buteo jamaicensis*) was excluded from the analyses because many of the vocalizations consisted of flying birds, whereas the exclusion of the hummingbirds was mainly due to difficulties in species identification of vocalizations during the acoustic monitoring.

### 2.5 Analyses

We followed the approach proposed by Moritz et al. (2008), and further developed by Tingley and Beissinger (2009), in which single‐season occupancy models that take into account differences in detectability among two time periods (*eras*) were used to compare the elevational profiles of the bird species. One advantage of this approach is that it may also control for differences in methodologies that could confound our comparisons (Moritz et al., 2008). Following the standard maximum likelihood hierarchical approach introduced by MacKenzie et al. (2002), our models contain a sampling level describing the probability of detection conditioned on occupancy (*p*), and an underlying biological level describing the probability (ψ) that a site is occupied. Because the contemporary surveys were not conducted at the same sampling sites of the historical surveys, we used an unpaired‐site model (Tingley & Beissinger, 2009) where data from all sites from both periods are used in a single‐season occupancy model, and both “*era*” and “*elevation*” are used as covariates influencing both occupancy and detection parameters. An example equation of the unpaired occupancy model can be described as:

Biological level—Occupancy (ψ)
  $$\text{logit}\left(\psi \right)=\beta_{0}+\beta_{1}era+\beta_{2}elevation+\beta_{3}elevation^{2}$$
Sampling level—Detectability (*p*)
  $$\text{logit}\left(p\right)=\beta_{4}+\beta_{5}era$$

To estimate elevational profiles of occupancy (ψ) for each species, we constructed a set of competing hypothesis of how occupancy changed over *era* and *elevation* (Table S1). *Era* is a covariate factor indicating the time period of surveys (historical and contemporary), whereas *elevation* is a standardized continuous covariate represented by a linear (*elev*) and a quadratic (*elev + elev* ^2^) function. We tested eight parameterizations of (ψ) by combining these covariates along with interaction terms and a null model (intercept‐only). The eight occupancy models were each run with each covariate separately in the detection function resulting in a total of 32 models per species (Table S1). Combinations of the covariates along with interaction terms were considered for the detection function, but exploratory analyses indicated problems with model convergence, so we opted to include covariates in the simplest form to reduce model set complexity (Burnham & Anderson, 2002). We compared models using AIC, and we estimated occupancy profiles across the range of elevations sampled by model‐averaging all models with ΔAIC <2.0. All models were fitted using the package Unmarked in R (Fiske & Chandler, 2011). Occupancy‐elevation profiles were created for each species and can be found in Fig. S1 along with cumulative AIC weights and model‐averaged occupancy‐elevation profiles for historic (blue) and current (gray) periods based on model‐averaging of models with ΔAIC <2.0. We also used the Wilcoxon signed‐rank test to compare detection probabilities between *eras* to determine which *era* had the highest average detection probability.

**Figure 2:**
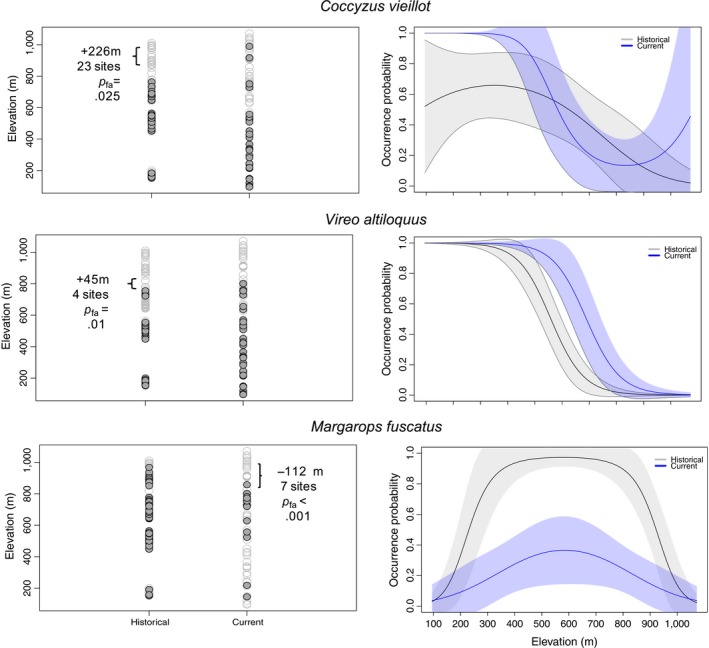
Example of elevation profiles of upward range expansion (*Coccyzus vieillot* and *Vireo altiloquus*) and downward range contraction (*Margarops fuscatus*). Shown are occupied (dark gray) and unoccupied (empty circles) sites, probability of false absence (*p*_fa_), and model‐averaged distribution‐elevation profiles

To estimate temporal shifts in the lower and upper range limits for each species, we followed the approach developed by Moritz et al. (2008). First, we compared the naïve elevation profile of each species to determine how many sites, at both the upper and lower limits of the range, a species was detected in one era, but not in the other. Then, we estimated the probability that the species was present at all of those sites and escaped detection (probability of false absence—*p*_fa_). To estimate *p*_fa_, we first estimated detection probabilities (p*) for each site by model‐averaging model‐specific *p* estimated from models with ΔAIC <2.0. We then calculated *p*_fa_ by multiplying 1 − p* to all sites where a species was detected in one era, but not the other, and then obtained the product of these values across these sites (Figure 2). Differently from Moritz et al. (2008), we considered all range limit shifts with *p*_fa_ < .05 as statistically significant.

## 3 RESULTS

Forty‐two bird species were detected in this study (Table S2), comprising 42% of the reported avifauna inhabiting the LM (Wunderle & Arendt, 2011). Thirty‐seven species were observed during the historical surveys, in which the most widespread species were the Scaly‐naped Pigeon (*Patagioenas squamosa*,* n* = 103/105 sites) and the Bananaquit (*Coereba flaveola*,* n* = 101/105), whereas six species, for example, the Bridled Quail‐Dove (*Geotrygon mystacea*) and the Puerto Rican Vireo (*Vireo latimeri*) were detected at just one site. Similarly, 33 species were detected in the acoustic monitoring, including two rare and endangered species, the Puerto Rican Sharp‐shinned Hawk (*Accipiter striatus venator*) and the Puerto Rican Broad‐winged Hawk (*Buteo platypterus brunnescens*), and one rare endemic of the Caribbean region Lesser Antillean Pewee (*Contopus latirostris blancoi*) not detected in the March–June 1998 sampling period. The most widespread species in the contemporary data set were the Bananaquit (59/60 sites) followed by the Puerto Rican Bullfinch (*Loxigilla portoricensis*, 57/60 sites) and the Puerto Rican Spindalis (*Spindalis portoricensis*, 57/60 sites), whereas six species, for example, Adelaide's Warbler (*Setophaga adelaidae*) and the Puerto Rican Broad‐winged Hawk, were detected at just one site.

Of the 26 species detected in both time periods, we tested for range shifts in the 20 species that had sufficient data for the occupancy analyses (Table 1). The Bananaquit was included in Table 1, but because it occurred in all but one site, we did not create an occupancy model. The covariate *era* was present in the best occupancy models for most species (*n* = 16/20) providing quantitative evidence of distributional changes between the two time periods. Furthermore, the covariate *elevation* was included in the most parsimonious models for all species in both eras except for the Ruddy Quail‐Dove (*Geotrygon montana*). A decrease in bird occurrence with elevation was the most common pattern in both times periods (*n* = 11) followed by a pattern of high bird occurrence at middle elevations (*n* = 8). Species detectability was, in general, higher for the contemporary period than for the historical period (Wilcoxon test, *W* = 4.5, *p *=* *.007).

**Table 1:** Summary of elevation changes for 21 species of forest birds inhabiting the Luquillo Mountains. Displayed are as follows: (1) American Ornithologists' Union four‐letters alpha codes for bird species; (2) average detectability per site for historical [*p* (H)] and contemporary [*p* (C)] periods; (3) historical elevation range; (4) changes in upper (U) and lower (L) range limit (bold text indicates significant shifts resulting from the *p*_fa_ tests); (5) the best supported form of the occupancy model (*Elev* = elevation; NA = not analyzed); (6) the cumulative Akaike's Information Criterion weight for all models with those terms (weight)

ID	Code	Species	*p* (H)	*p* (C)	Historical elevation range (m)	Range limit change (m)	Best occupancy model	Weight
*Range expansion*								
1	PRLC	*Coccyzus vieillot*	.30	.30	152–763	**+223 U**	*Era* × *Elev* ^2^	0.67
2	BWVI	*Vireo altiloquus*	.68	.93	151–755	**+45 U**	*Era* + *Elev*	0.53
3	EWWA	*Setophaga angelae*	.04	.82	660–980	+46 U +121 L	*Elev* ^2^	0.55
4	PRSO	*Megascops nudipes*	.15	.35	200–776	+133 U +105 L	*Era* × *Elev*	0.28
5	RUQD	*Geotrygon montana*	.16	.16	184–660	**+329 U** **+ 66 L**	*Era*	0.46
6	PRTA	*Nesospingus speculiferus*	.33	.70	152–968	**+78 U**	*Era* × *Elev* ^2^	0.57
7	PRFL	*Myiarchus antillarum*	.15	.15	151–564	+197 U	*Era* × *Elev*	0.59
8	AMRE	*Setophaga ruticilla*	.38	.51	647–684	**+67 U** **+408L**	*Era* × *Elev* ^2^	1.00
9	PRWO	*Melanerpes portoricensis*	.36	.51	151–990	+47 U	*Elev* ^2^	0.35
10	ANEU	*Euphonia musica*	.06	.05	641–684	+116 U +**523 L**	*Era* × *Elev* ^2^	0.73
11	PRSP	*Spindalis portoricensis*	.25	.86	152–979	+93U	*Era* × *Elev*	0.51
12	MACU	*Coccyzus minor*	.11	.12	181–190	+55U +83L	*Elev*	0.31
13	PROP	*Icterus portoricensis*	.16	.06	153–183	+239	*Era* + *Elev*	0.25
*Range contraction*								
14	PETH	*Margarops fuscatus*	.70	.70	152–968	**−112 U**	*Era* × *Elev* ^2^	0.54
15	ZEND	*Zenaida aurita*	.17	.17	152–776	**−480 U**	*Era* × *Elev* ^2^	0.65
*No change*								
16	PRBU	*Loxigilla portoricensis*	.49	.89	151–1,011	No change	*Era* × *Elev* ^2^	0.37
17	PRTO	*Todus mexicanus*	.32	.68	152–993	+4 U	*Era* + *Elev* ^2^	0.23
18	SNPI	*Patagioenas squamosa*	.76	.76	151–1,011	No change	*Era* × *Elev*	0.83
19	RLTH	*Turdus plumbeus*	.58	.58	151–763	+9 U	*Era* × *Elev* ^2^	0.34
20	GRAK	*Tyrannus dominicensis*	.19	.19	151–999	No change	*Era* × *Elev* ^2^	0.98
21	BANA	*Coereba flaveola*	NA	NA	151–1,011	No change	NA	NA

**Figure 3:**
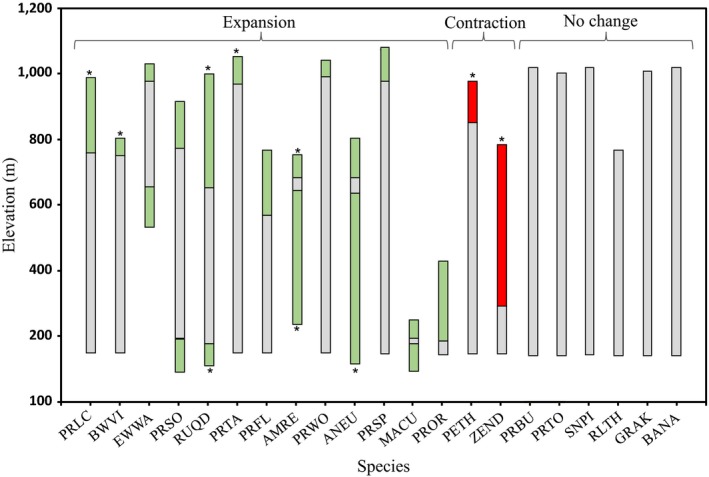
Elevational range changes for 21 species of forest birds inhabiting the Luquillo Mountains (see Table 1 for species' alpha codes). Significant (*p*_fa_ < .05) shifts are marked with (*) at the range limit. Range expansions are colored in green, whereas range contractions are colored in red for expansions and blue for contractions, whereas nonsignificant shifts are colored green (Table 1)

**Figure 4:**
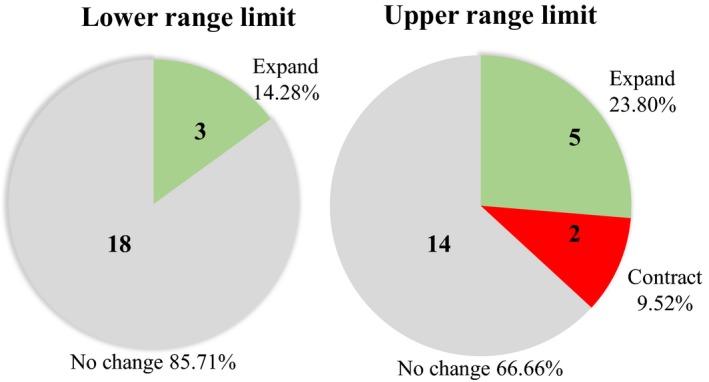
Number and proportion of upper and lower range shifts for 21 species of forest birds inhabiting the Luquillo Mountains. Pie charts display the proportions of range limits that exhibited significant expansions (green), contractions (red), or no significant change (gray). Numbers represent the number of individual shifts observed in each category

We observed an overall pattern of no significant change in the range limits for most species (*n* = 13/21) (Figure 3). Of the eight species (38%) that had a significant shift in their range, six expanded and two contracted (Table 1, Figures 3 and 4). Of the six species that expanded their range, the most common pattern was an increase in their upper range limit (*n* = 5). We detected a significant increase in the upper range limit in five species (23, 80%, mean = 148 m) and a significant decrease in two species (9, 52%, mean = 296 m). A significant increase in lower range limit was detected in three species (14, 28%), of which all increased (mean = 332 m).

## 4 DISCUSSION

The elevational distribution of eight of 21 species changed significantly over a span of 17 years. We hypothesize that the rapid response by these species is driven by increasing temperature in Puerto Rico, rather than anthropogenic disturbances given that the study site has been protected for >100 years. The elevation distributions of six bird species expanded, whereas the ranges of two species contracted. Overall, these range shifts concur with other studies that have revealed a common pattern of an upward shift in elevation for many species (Table S3), usually associated with the expansion of the upper range limit as a result of the effect of climate change (Parmesan & Yohe, 2003).

Comparison of avian elevational distributions changes for birds across sites and studies is challenging because there are relatively few studies on bird range shifts, especially in the tropics (Table S3). Furthermore, these studies vary in the extent of temperature change, the time scale of each study, and differences in the elevational gradient. Such variation results in disparate humidity and precipitation regimes, and edaphic effects, which in turn affect vegetation associations and, therefore, avian resources such as shelter, food, and water, thereby dictating consequential variants of species richness, composition, abundance, reproduction, and many other biological and ecological parameters.

Changes in temperatures reported for Puerto Rico and the LM fall within the temperature ranges of studies that detected both significant and nonsignificant changes in bird distributions (Table S3). Similarly, both significant and nonsignificant changes were found during short time scales (<20 years), similar to the period of our study (Table S3). The extent of the elevational gradient in our study is relatively small compared to other studies, but both significant and nonsignificant changes were found over larger elevational gradients (Table S3). The only other study that has tested for elevational distributions changes in birds in tropical island ecosystem found a strong pattern of upslope shift in birds (Freeman & Class Freeman, 2014). Therefore, there are no clear patterns among these variables and the ability to detect significant range shifts in birds, and more studies are needed to understand the drivers of range shifts in birds.

Using occupancy models that consider differences in detectability associated with the sampling process in the two time periods, we provided an unbiased comparison of distributional changes along a tropical mountain elevation gradient. One limitation of our study, and other studies on the effects of climate change on species distributions (Chen et al., 2009; Chen, Hill, Shiu et al., 2011; Forero‐Medina, Terborgh, Socolar, & Pimm, 2011; Raxworthy et al., 2008), is that it only included two points in time. Nevertheless, our study along with these previous studies provides essential baseline information on the response of species distributions to climate change in tropical montane forests. Although our results should be considered with caution, they do indicate a significant and rapid shift in the ranges of eight species in a protected tropical mountain reserve. Furthermore, we provide a quantitative documentation of the distributions of 21 species that can guide future research on the impacts of climate change.

Another important consideration for our study is that although the LM have not suffered deforestation for more than 100 years, they have been exposed to at least two severe hurricanes during the last 50 years (Georges, 1998; Hugo, 1989). Body mass, diet, reproduction, survival rates, abundance, and distribution of birds have been documented to change (Arendt, 2000; Waide, 1991), even after approximately 2 years' posthurricanes (Arendt, 2006; Wunderle, 1995). For instance, immediately after the Hurricane Hugo, there was a dramatic decrease in abundance of nectarivorous and frugivorous bird species and an increase in abundance of insectivores and omnivores. Nevertheless, the abundance of all species returned to prehurricane levels in the next breeding season (Waide, 1991). Because of the long‐term impact caused by hurricanes, we chose to use the historical sample of March–June 1998, which was more than 10 years after Hurricane Hugo and more than 2 months before Hurricane Georges, to minimize extreme variations in bird distribution associated with these hurricanes.

The relatively rapid distributional change over 17 years in one of the best‐protected forests in Puerto Rico may have significant ecological consequences for the dynamics of the LM, such as cascading effects and disruption of trophic interactions, because birds are the main top predators and seed dispersers on the island (Reagan & Waide, 1996). For instance, one important top predator in Puerto Rico, the Puerto Rican Lizard‐Cuckoo (*Coccyzus vieilloti*) is found primarily in lowland (ψ > 0.50 below 600 m), and is currently expanding its distribution to high‐elevation areas, which include the elevation zone of the most endangered frog species in the LM (M. Campos‐Cerqueira and T. Mitchell Aide, unpublished data). This top predator, along with the Pearly‐eyed Thrasher (*Margarops fuscatus*—range contraction) and the Puerto Rican Tanager (*Nesospingus speculiferus*—range expansion) is important in the ecological dynamics of the LM as they exert top–down pressure on the Anolid lizards and Eleutherodactylus frogs that in turn, constitute the largest component of the diurnal and nocturnal biomass of all vertebrates in Puerto Rico (Pérez‐Rivera, 1995; Reagan & Waide, 1996; Stewart, 1995). In addition, our results indicate that two invertivorous migrants the Black‐wiskered Vireo (*Vireo altiloquus*) and the American Redstart (*Setophaga ruticilla*) may be changing their elevational distribution. The range shifts of these two birds may suggest that the distribution of invertebrate biomass may also be changing. This shift in invertebrate biomass was recently observed by other researchers in the LM (Brad Lister, in litt; Schowalter, Willig, & Presley, 2017). A similar pattern was also found in Jamaica where the spatial distribution of migratory warblers was correlated with insect biomass and not abundance of predators and resident competitors or with vegetation characteristics (Johnson & Sherry, 2001).

Another significant range shift that may have important conservation implications is an elevational contraction of the Pearly‐eyed Thrasher. This supertramp species (Arendt, 2004, 2006) is a well‐known predator on the eggs, young, and even adults of other birds and thus, the range contraction of this species may alleviate predation pressure on other species inhabiting high‐elevation areas. In addition, the thrasher is known to compete for cavity nests with the endangered Puerto Rican Parrot (*Amazona vittata*) (Arendt, 2000; Snyder, Wieley, & Kepler, 1987). The critically endangered Puerto Rican Parrot BirdLife International (2016) suffered a population decline of 2000 (1937) to 13 individuals (1975), had an estimated population of 16–20 birds by 2013 in the LM (White, Collazo, Dinsmore, & Llerandi‐Roman, 2014), and historically occurred along the entire elevational gradient (Snyder et al., 1987). Thus, any distributional changes by the thrasher may influence the biological and ecological interactions between these two nest‐site competitors, negatively affecting both species' reproductive success, but especially that for the parrot in the lowlands, while decreasing competition at high elevations. Indeed, evidence suggests that thrasher abundance is decreasing at mid‐ and lower elevations in the LM (Arendt, 2006: fig. 1.18).

Among the other bird species that showed significant range expansion, the Ruddy quail‐Dove (*Geotrygon Montana*) is widespread in the lowlands of the Neotropics (although occurring up to 2,600 m), and is known to feed on fruits, insects and seeds, as does the Antillean Euphonia (*Euphonia musica*), which inhabits at least 16 islands in the Caribbean from Hispaniola in the Greater Antilles to Grenada, Lesser Antilles (Arendt, 2006: appendix 1, p. 375). It feeds mainly on mistletoe but as are many Caribbean birds, it is a dietary generalist and forages on a wide variety of plant material, including fruits but occasionally feed on invertebrates (Pérez‐Rivera, 1991). The Puerto Rican Tanager feeds mostly on palm fruits, ants, and species from the genus *Cecropia*. However, they occasionally eat spiders, lizards, and frogs (Pérez‐Rivera, 1995). Together, the range expansion of these two potential seed‐dispersers species may influence vegetation's abundance, distribution, and assemblages in high‐elevation areas. The only other species that exhibited range contraction was the Zenaida Dove (*Zenaida aurita*), which feeds mainly on seeds (especially of grasses and herbs) and is a typical lowland species that may have taken advantage of hurricane‐induced openings and clearings along many of the abandoned roads inside the LM, which are now overgrown with wood vegetation.

Our study provides a quantitative description of rapid elevational shifts in bird species in a tropical mountain, and a quantitative baseline for future studies of these and other species, not only in the tropics but worldwide. Because elevational distribution affects species extinction risk (Blake & Loiselle, 2015; Gage, Brooke, Symonds, & Wege, 2004; Sekercioglu, Schneider, Fay, & Loarie, 2008), studies that provide a detailed quantitative description of species elevational range are essential to improving predictions for species vulnerability to climate and land‐use changes. In addition, almost half (49%) of the resident birds detected in this study are endemic species and, therefore, detailed information about changes in their distributions is needed to assess their vulnerability to climate change. From a conservation perspective, although this is the second study to quantitatively assess forest bird distribution in the LM along an elevational gradient (see Pagán, 1995), it is the first study to use occupancy modeling to provide a quantitative elevational profile of the birds in the LM, which can now be used to improve conservation management. For instance, our study corroborates three previous studies documenting that the endemic and now threatened Elfin‐woods Warbler (*Setophaga angelae*), has a restricted elevational distribution now occurring mainly between 600 and 900 m, within the Palo Colorado forest (Anadón‐Irizarry, 2006; Arendt, 2006; Campos‐Cerqueira & Aide, 2016), even though the original description of the species' habitat, suggested a strong association with elfin woodland above 800 m (Kepler & Parkes, 1972).

Overall, acoustic monitoring provides a more robust method to detect species (Celis‐Murillo, Deppe, & Ward, 2012; Wimmer, Towsey, Roe, & Williamson, 2013), given general higher detection probabilities and lower uncertainty in the occupancy estimates. Besides an increase in detectability, acoustic monitoring has many other advantages: (1) Recordings can be permanently stored, functioning as “museum vouchers,” allowing future comparisons and analyses; (2) observer bias is minimized, as recordings can be accessed by different experts; (3) sampling can occur simultaneously along the entire gradient, minimizing temporal or spatial bias in the sampling process; and (4) acoustic monitoring can be used to better infer patterns of arrival and departure of migrants and temporal changes in avian reproduction as vocalizations are often a good signal of reproduction. Although these latter two parameters were recorded in the historic population surveys in LM, acoustic monitoring increases the precision of the empirical data because of the units' high‐quality recordings and cost‐effectiveness, that is, increased low‐cost spatiotemporal coverage. In contrast, traditional methods (e.g., distance sampling, mist nettings) allow an easier means of estimating species abundance than the acoustic monitoring and may provide other important biological information such as the assessment of active incubation patches, cloacal protuberances, commissures (gapes), all of which aide in sexing and aging birds in the hand. Moreover, the combination of different techniques may be the best solution to sampling the entire bird community, especially in regions of high species richness such as the tropics.

In this study, we have provided a quantitative description of rapid elevational shifts in bird species along a tropical mountain; presented data to confirm general impressions of bird distribution; and corroborate the level of threat for particular species. Our results further illustrate how acoustic monitoring provides an easy and powerful way to monitor animal populations along elevational gradients. We encourage studies that will test potential mechanisms driving these rapid bird shifts, and we encourage the establishment of long‐term monitoring projects around the globe to improve information of species distributions given the threat of climate change.

## AUTHOR CONTRIBUTIONS

MCC and TMA, together, designed the study. MCC processed audio recordings and performed modeling work. WJA performed point counts. MCC, JMW, WJA identified bird species in the recordings. MCC, JMW, WJA and TMA analyzed output data. MCC wrote first draft of the manuscript. All authors contributed to revisions of the manuscript.

## CONFLICT OF INTEREST

None declared.
