# Supplementary material for: Have bird distributions shifted along an elevational gradient on a tropical mountain?
Source: Ecol Evol. 2017 Oct 20;7(23):9914–24. doi: 10.1002/ece3.3520 (PMC5723601; doi:10.1002/ece3.3520)
Supplement: Supplementary file 1 [file ECE3-7-9914-s001.pdf]

A

1- Coccyzus vieillot

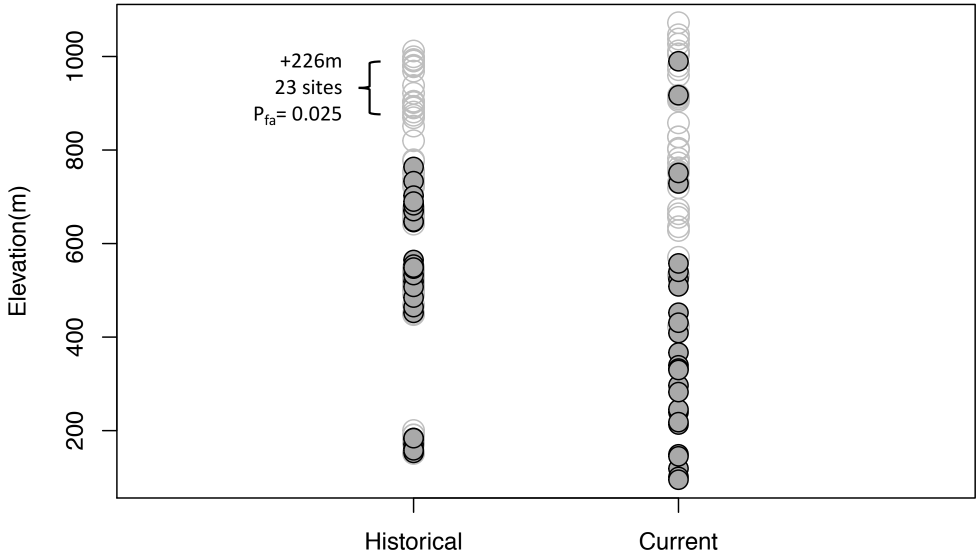

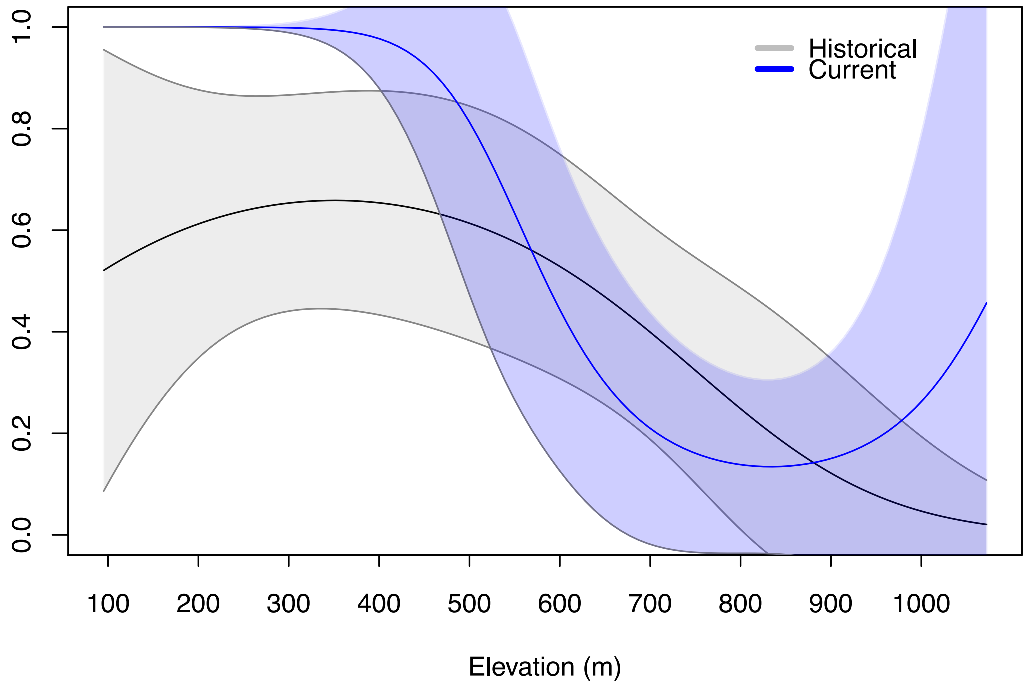

**Occupancy models**	**Cumulative AIC weight**
Constant	0
Elev	0.07
Elev+Elev^2	0.02
Era	0
Era+Elev	0.04
Era+Elev+Elev^2	0.02
Era*Elev	0.15
**Era*(Elev+Elev^2)**	**0.67**

Optimal historical= 351m
Optimal current= 95 – 331m

B

2 - Vireo altiloquus

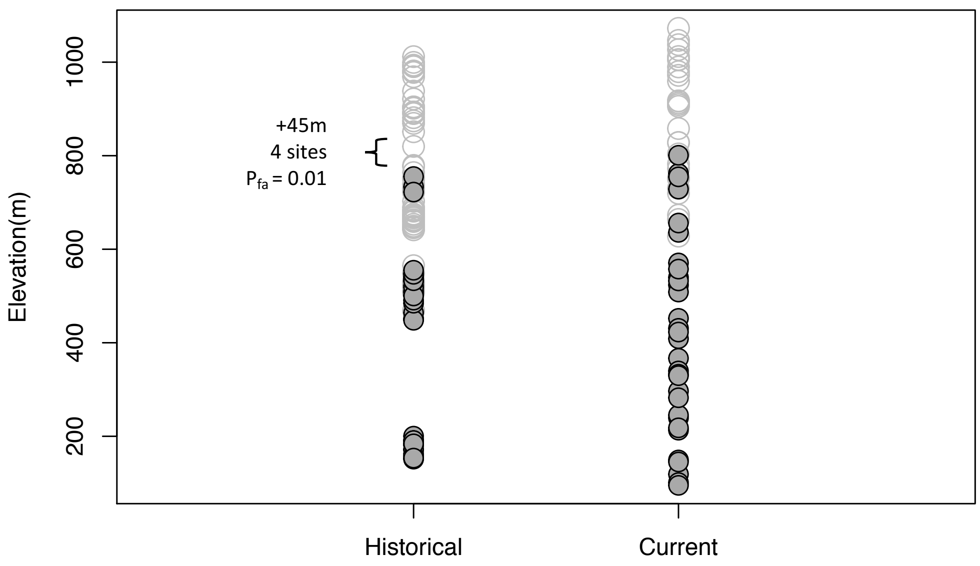

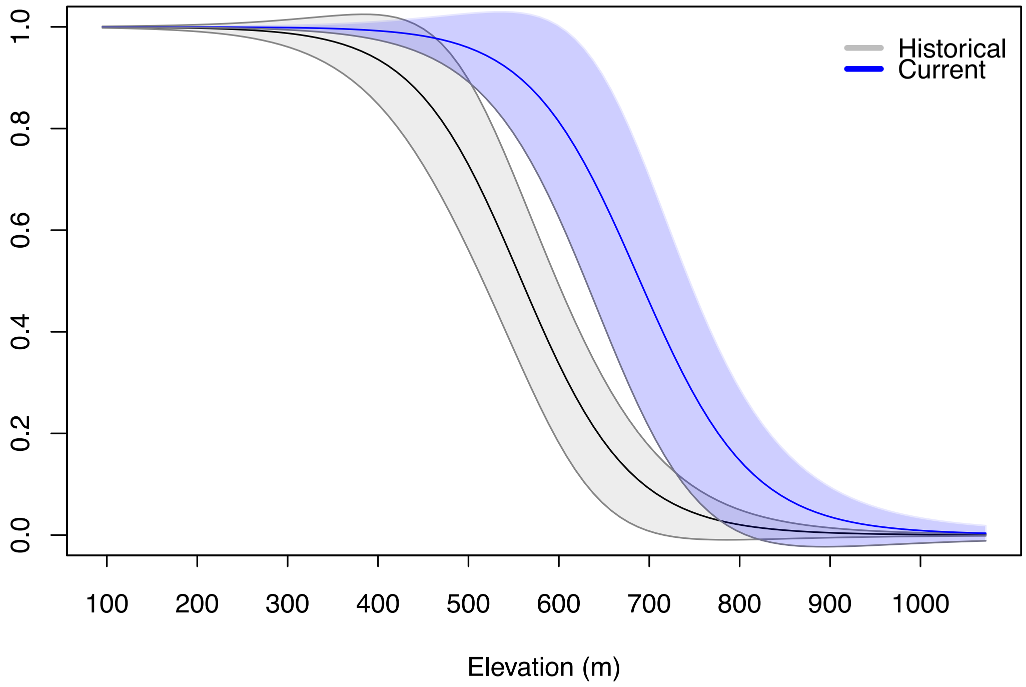

Occupancy models	Cumulative AIC weight
Constant	0
Elev	0.01
Elev+Elev²	0.00
Era	0
**Era+Elev**	**0.53**
Era+Elev+Elev²	0.23
Era*Elev	0.19
Era*(Elev+Elev²)	0.04

Optimal historical= 95 - 243 m
Optimal modern= 95 - 371m

C

3 – *Setophaga angelae*

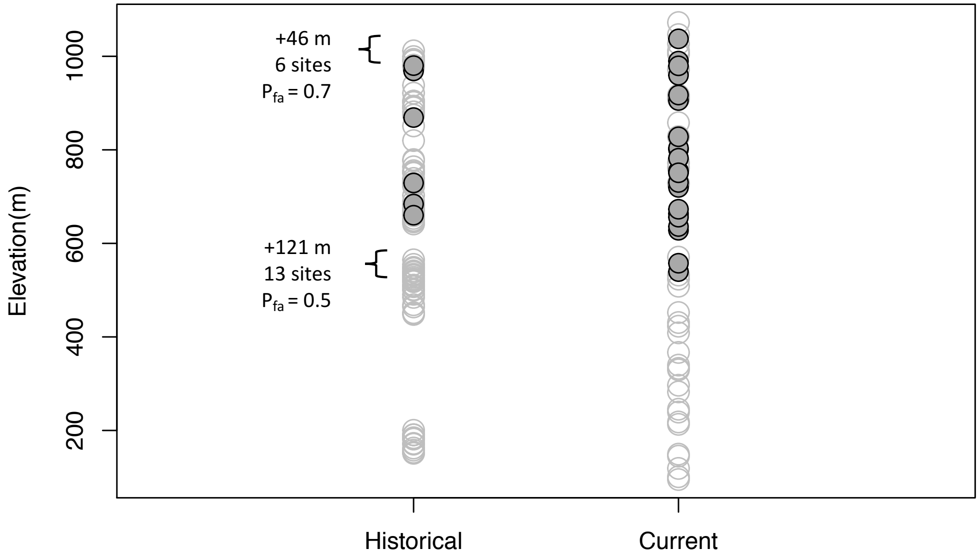

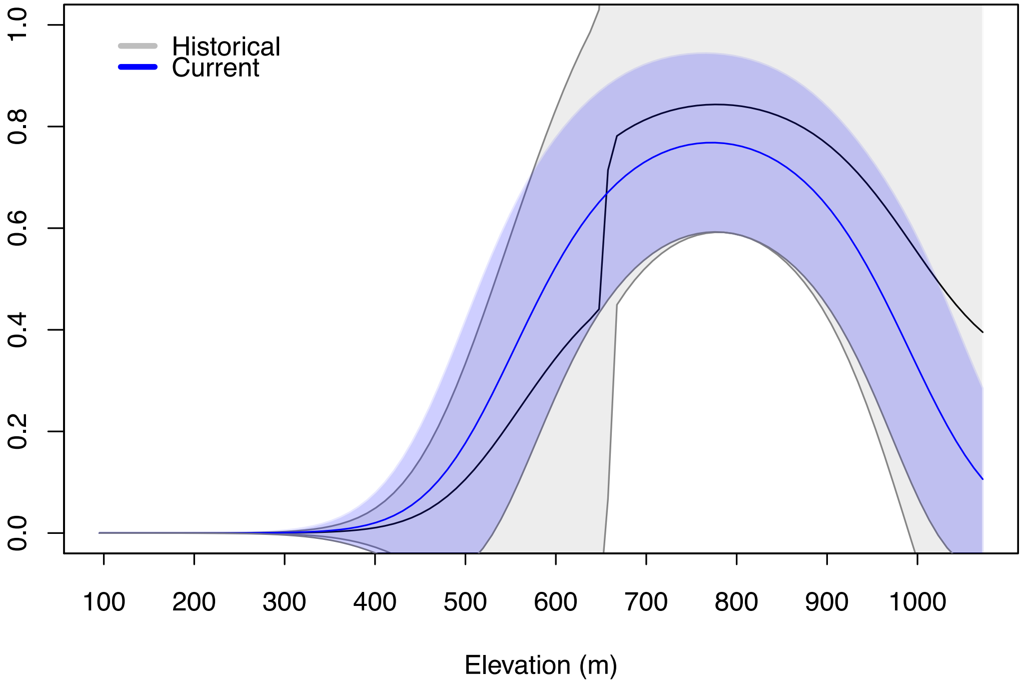

Occupancy models	Cumulative AIC weight
Constant	0
Elev	0
**Elev+Elev^2**	**0.55**
Era	0
Era+Elev	0.00
Era+Elev+Elev^2	0.20
Era*Elev	0.00
Era*(Elev+Elev^2)	0.25

Optimal historical= 736 - 815 m
Optimal modern= 756 - 785 m

D

4 – Megascops nudipes

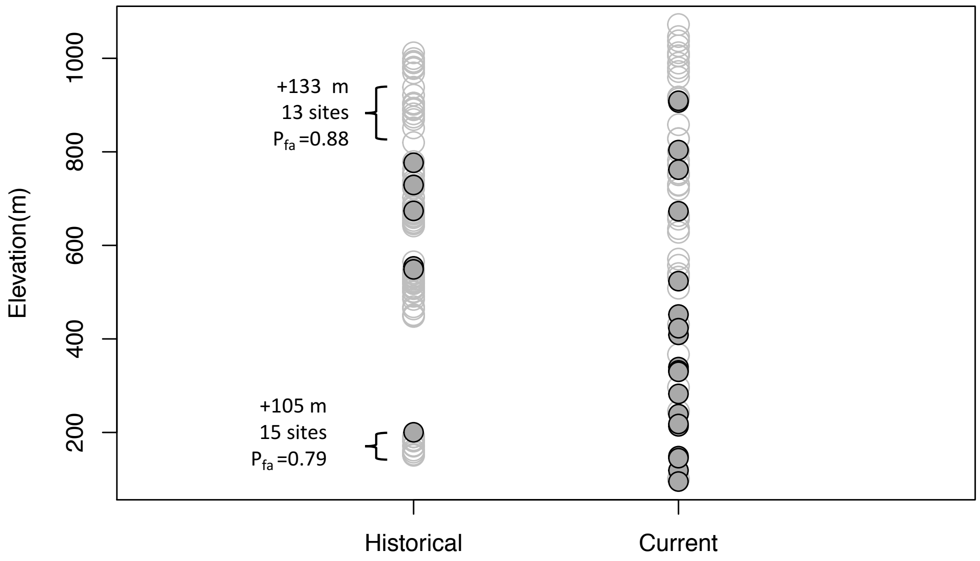

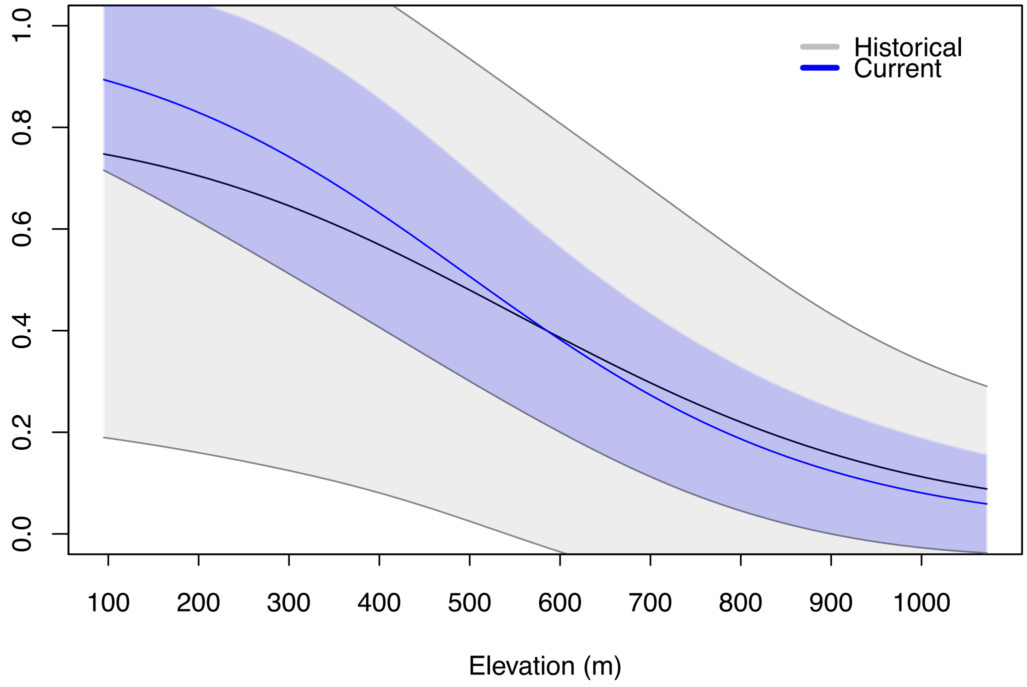

Occupancy models	Cumulative AIC weight
Constant	0
Elev	0.25
Elev+Elev^2	0.09
Era	0.01
Era+Elev	0.20
Era+Elev+Elev^2	0.09
**Era*Elev**	**0.28**
Era*(Elev+Elev^2)	0.08

Optimal historical= 95 m
Optimal modern= 95 m

E

5 – Geotrygon montana

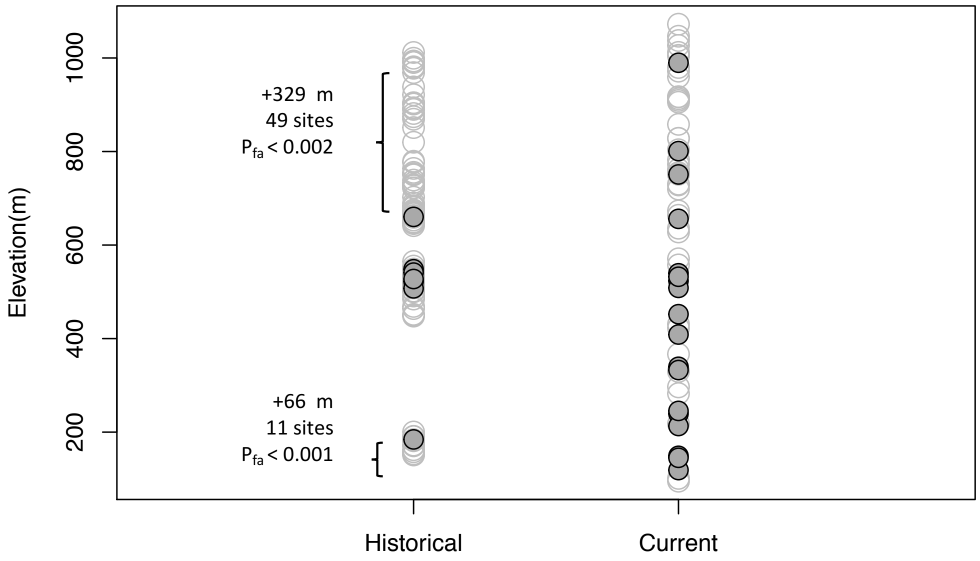

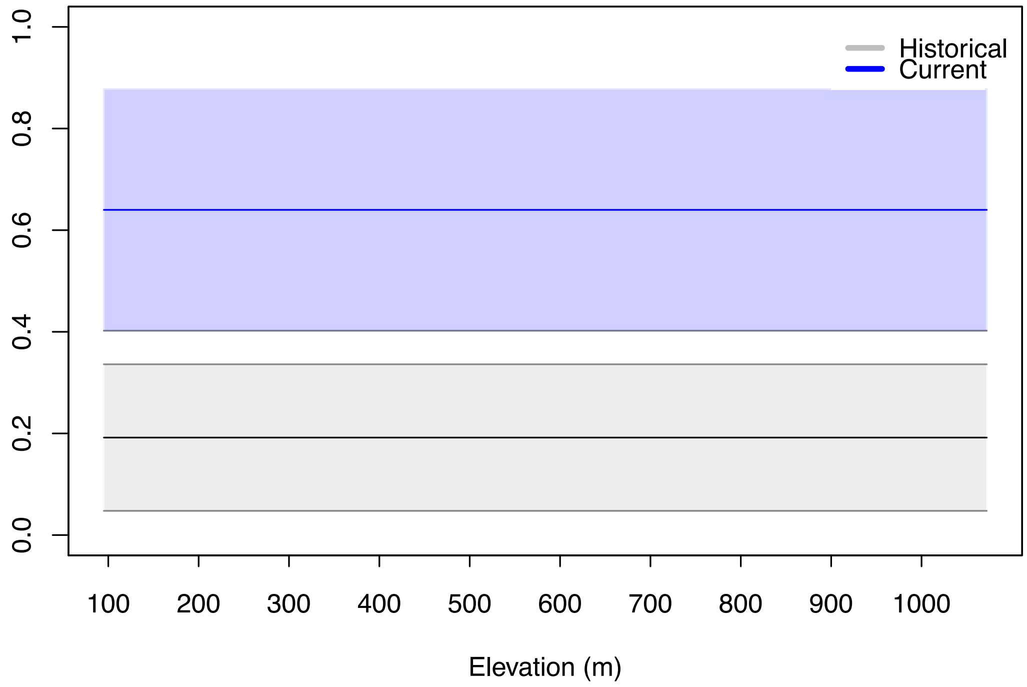

Occupancy models	Cumulative AIC weight
Constant	0.01
Elev	0.01
Elev+Elev^2	0.01
**Era**	**0.46**
Era+Elev	0.18
Era+Elev+Elev^2	0.18
Era*Elev	0.09
Era*(Elev+Elev^2)	0.07

Optimal historical= 95 - 1072 m
Optimal modern= 95 - 1072 m

F

6 – *Nesospingus speculiferus*

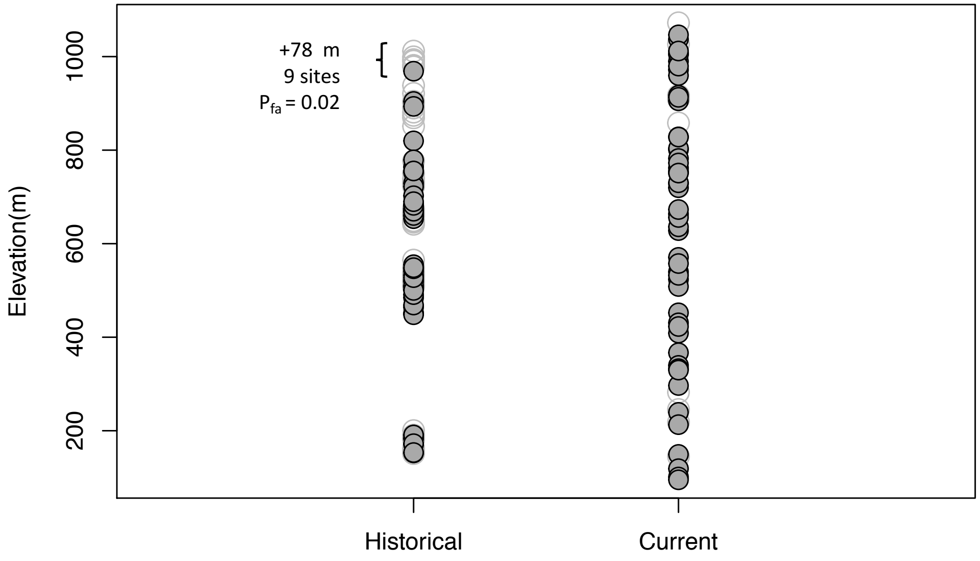

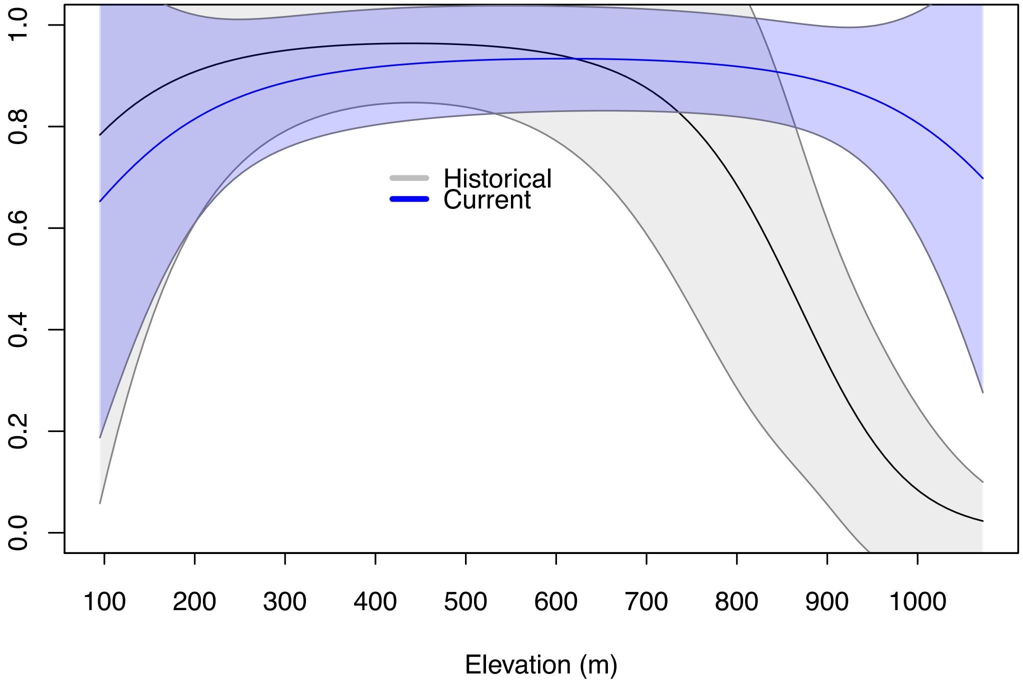

Occupancy models	Cumulative AIC weight
Constant	0.00
Elev	0.00
Elev+Elev^2	0.02
Era	0.00
Era+Elev	0.00
Era+Elev+Elev^2	0.17
Era*Elev	0.24
**Era*(Elev+Elev^2)**	**0.57**

Optimal historical= 331-539 m
Optimal modern= 963-1072 m

G

7 - *Myiarchus antillarum*

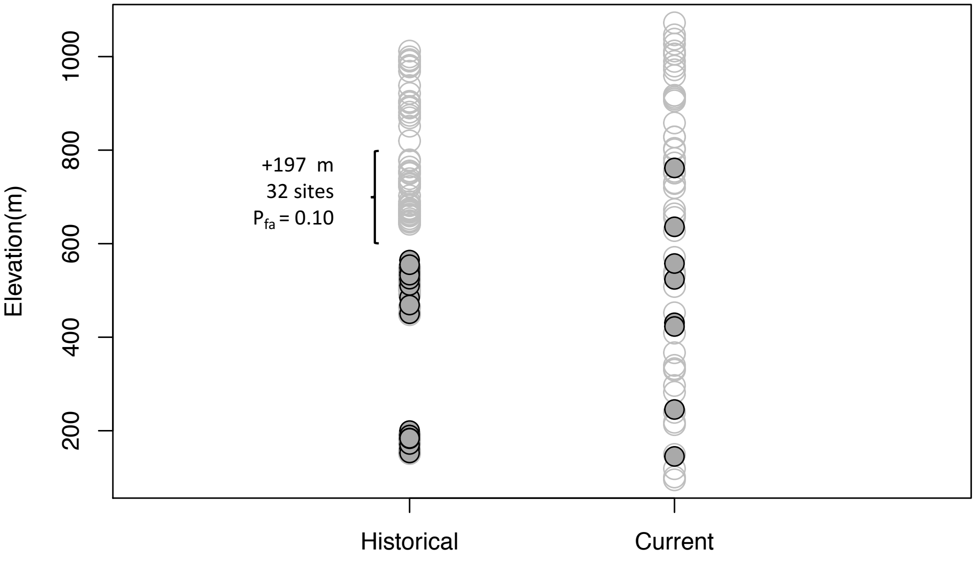

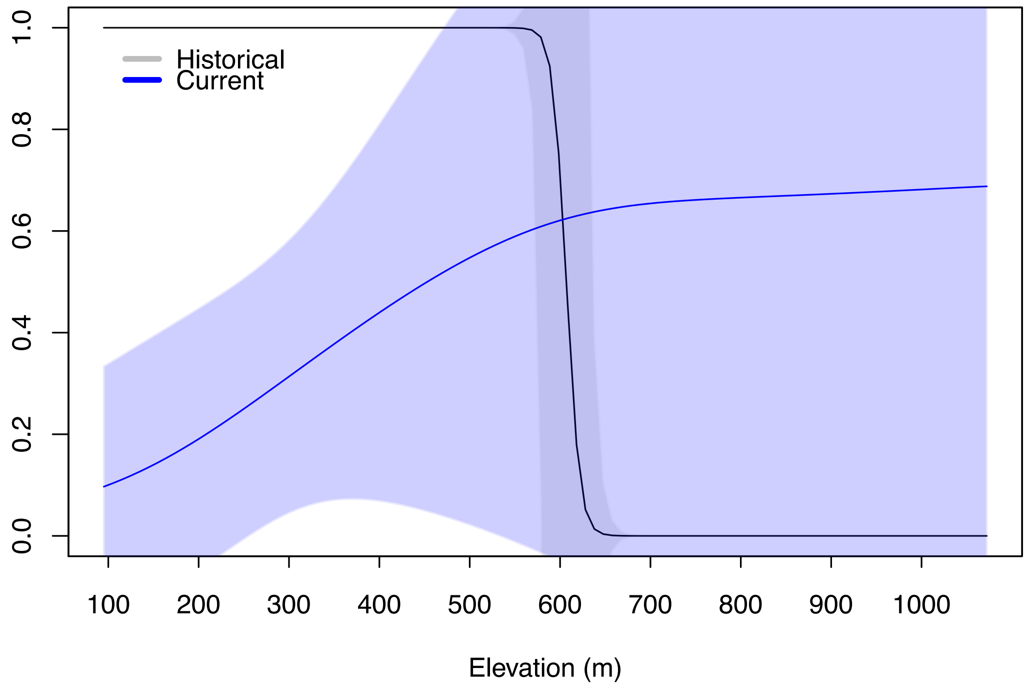

Occupancy models	Cumulative AIC weight
Constant	0.00
Elev	0.00
Elev+Elev^2	0.00
Era	0.02
Era+Elev	0.09
Era+Elev+Elev^2	0.06
**Era*Elev**	**0.59**
Era*(Elev+Elev^2)	0.24

Optimal historical= 95-568 m
Optimal modern= 1042-1072 m

H

8 – *Setophaga ruticilla*

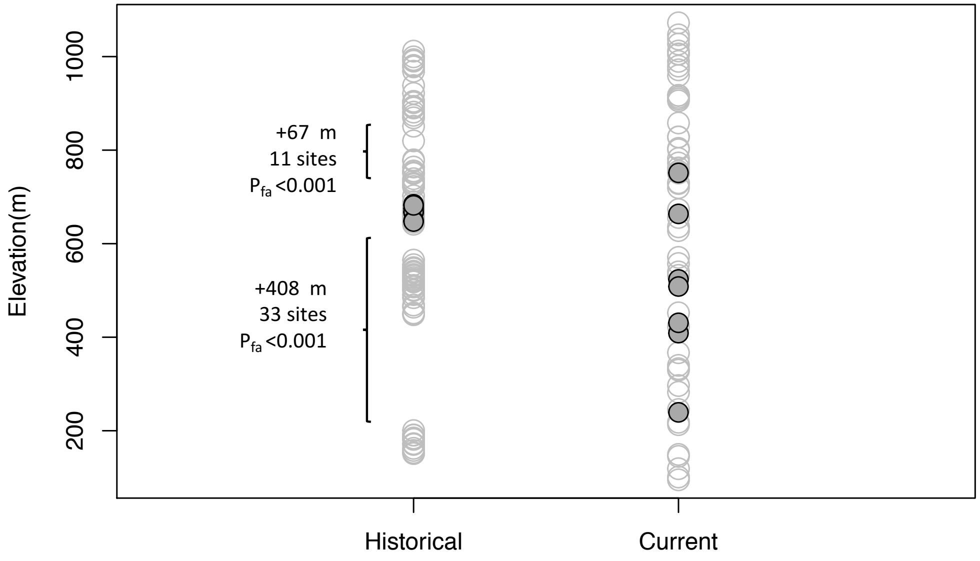

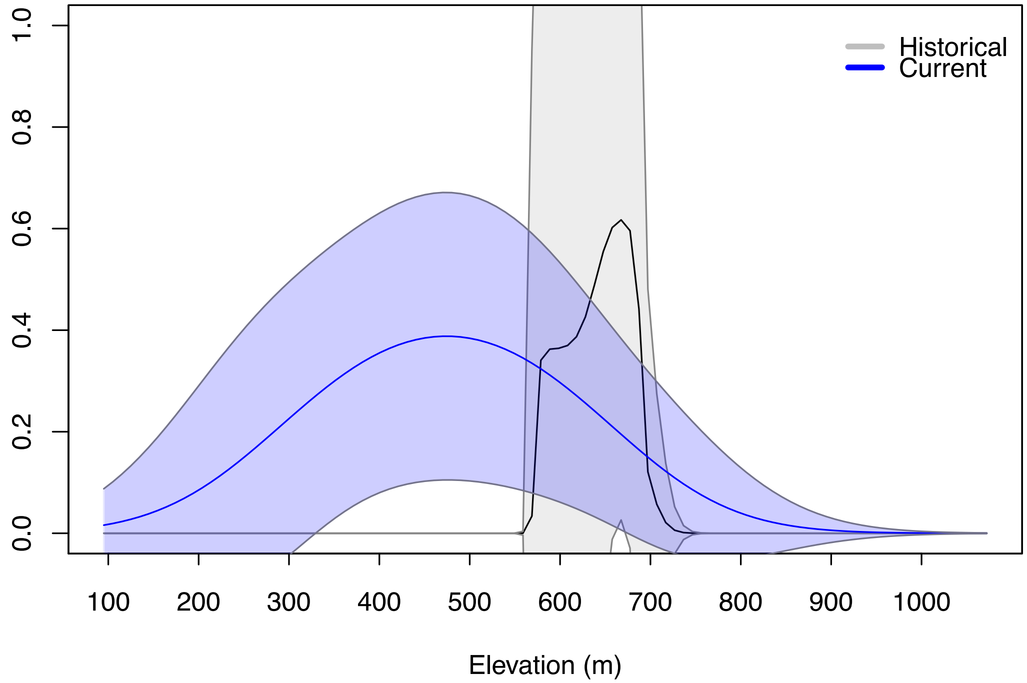

**Occupancy models**	**Cumulative AIC weight**
Constant	0.00
Elev	0.00
Elev+Elev^2	0.00
Era	0.00
Era+Elev	0.00
Era+Elev+Elev^2	0.00
Era*Elev	0.00
**Era*(Elev+Elev^2)**	**1.00**

Optimal historical= 667 m
Optimal modern= 460-489 m

I

9 – Melanerpes portoricensis

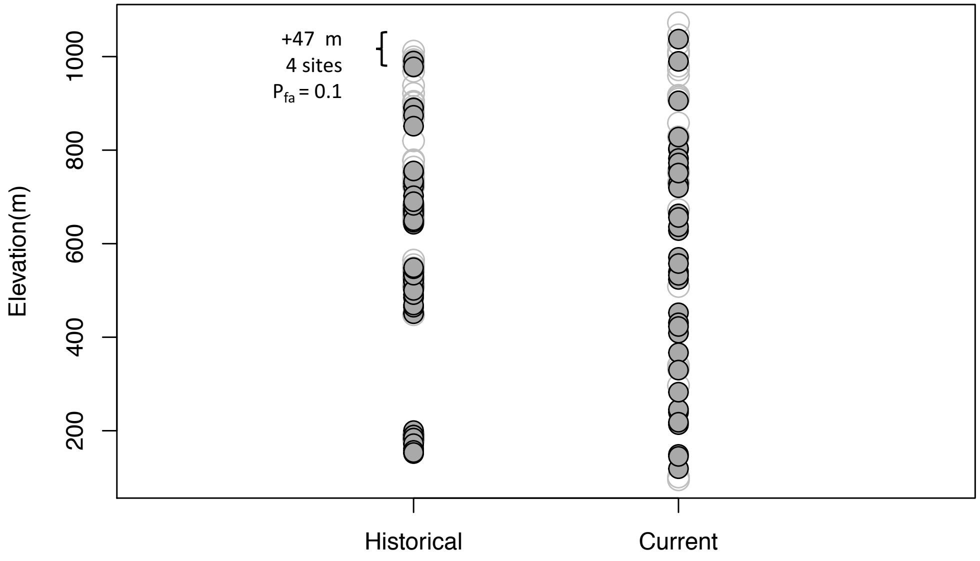

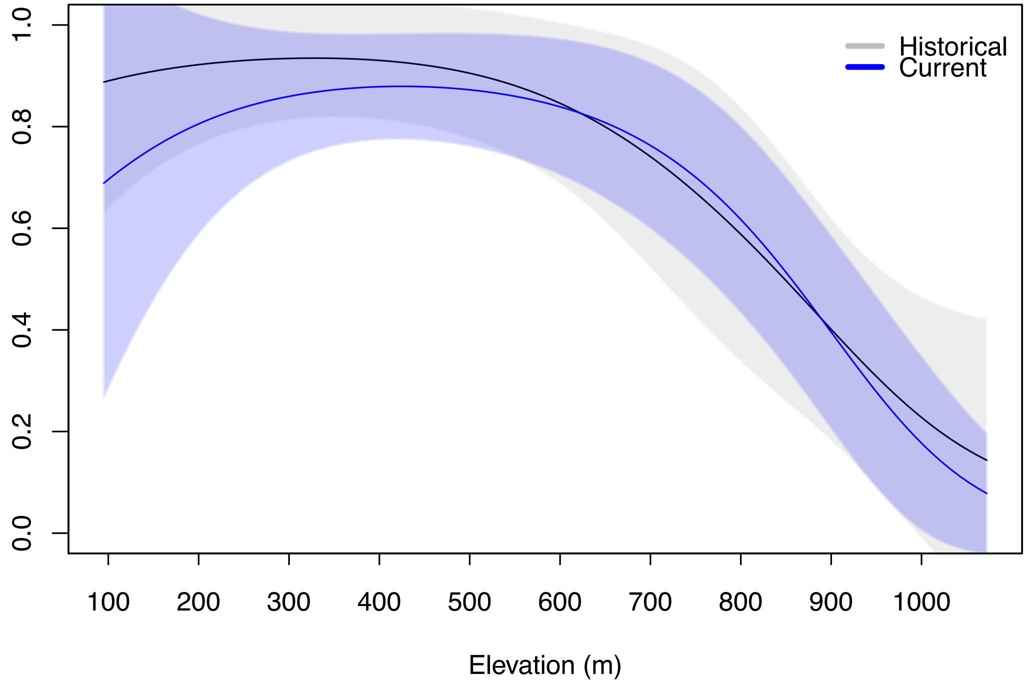

Occupancy models	Cumulative AIC weight
Constant	0.00
Elev	0.07
**Elev+Elev²**	**0.35**
Era	0.00
Era+Elev	0.03
Era+Elev+Elev²	0.18
Era*Elev	0.04
Era*(Elev+Elev²)	0.34

Optimal historical= 223-430 m
Optimal modern= 371-480 m

J

10 – *Euphonia musica*

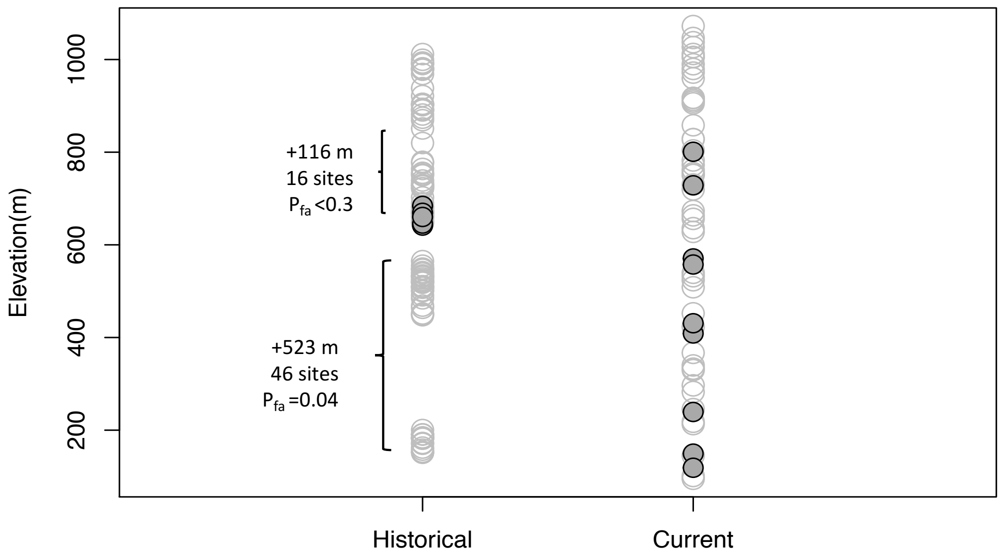

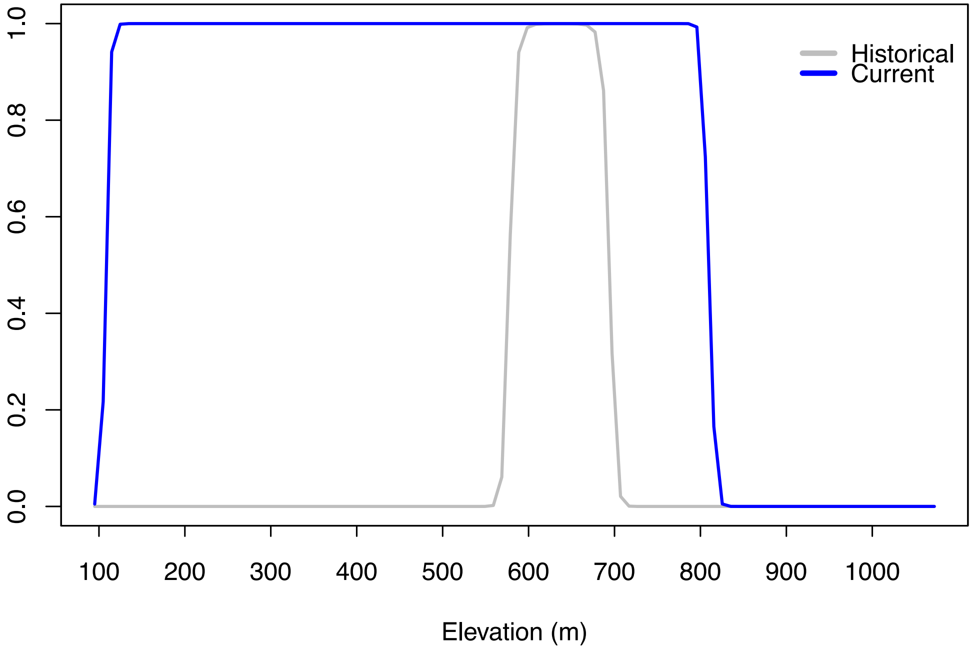

Occupancy models	Cumulative AIC weight
Constant	0.00
Elev	0.01
Elev+Elev^2	0.01
Era	0.00
Era+Elev	0.11
Era+Elev+Elev^2	0.07
Era*Elev	0.07
**Era*(Elev+Elev^2)**	**0.73**

Optimal historical= 608-667 m
Optimal modern= 124-785 m

K

11 – Spindalis portoricensis

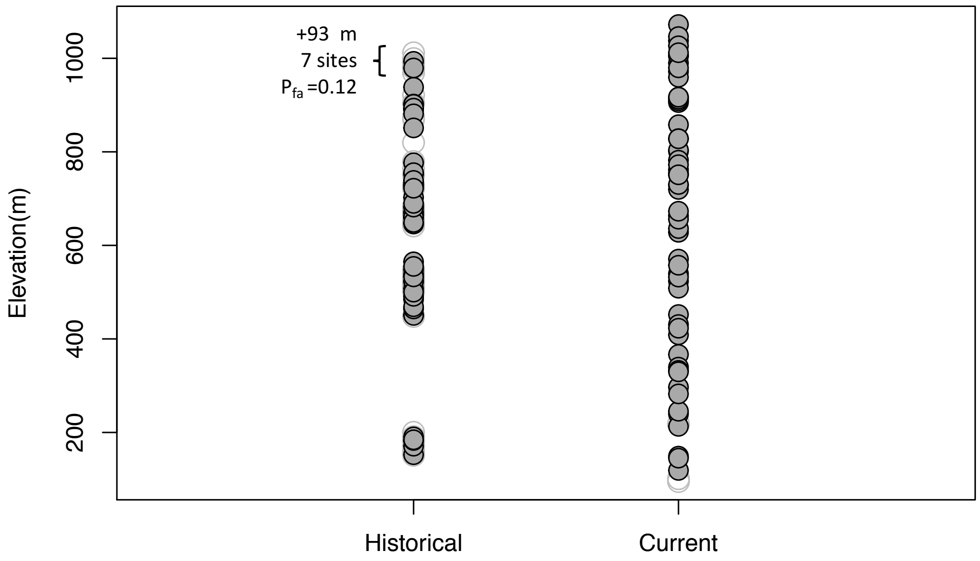

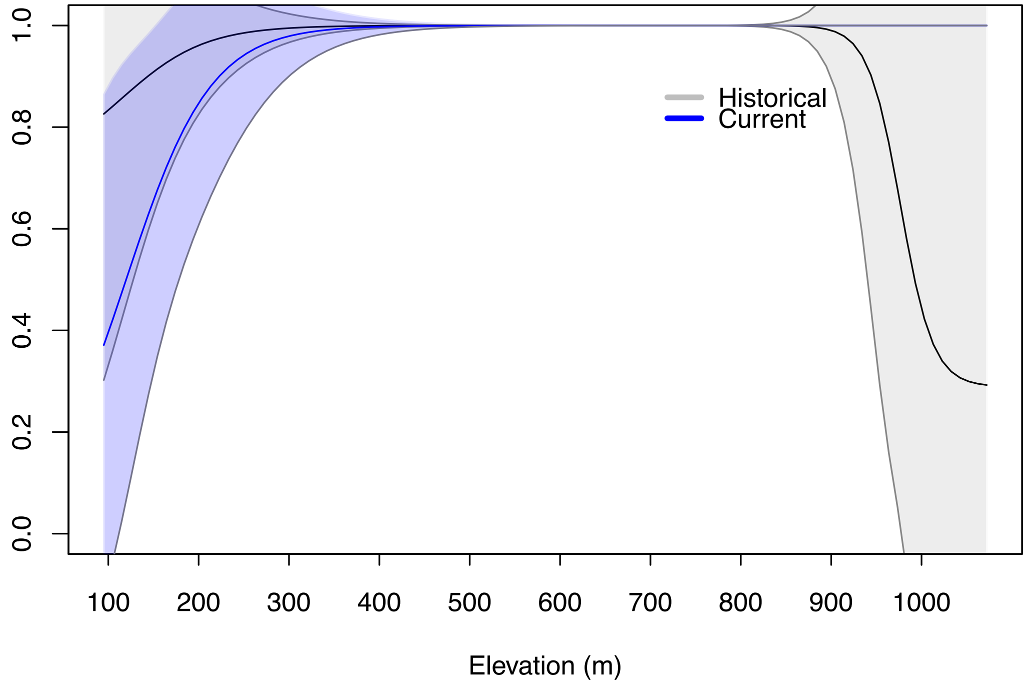

Occupancy models	Cumulative AIC weight
Constant	0.00
Elev	0.21
Elev+Elev^2	0.08
Era	0.00
Era+Elev	0.09
Era+Elev+Elev^2	0.03
**Era*Elev**	**0.51**
Era*(Elev+Elev^2)	0.07

Optimal historical= 302-884 m
Optimal modern= 371 -1072 m

L

12 – Coccyzus minor

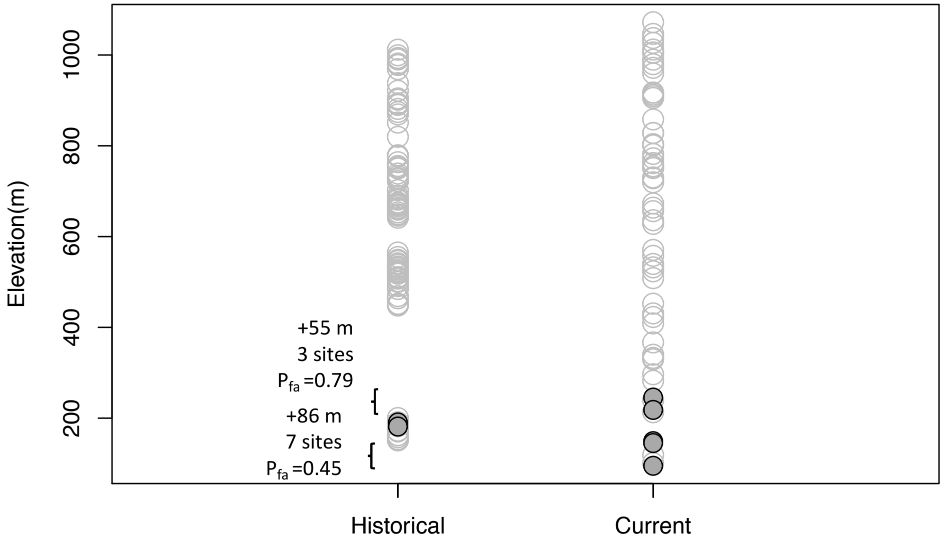

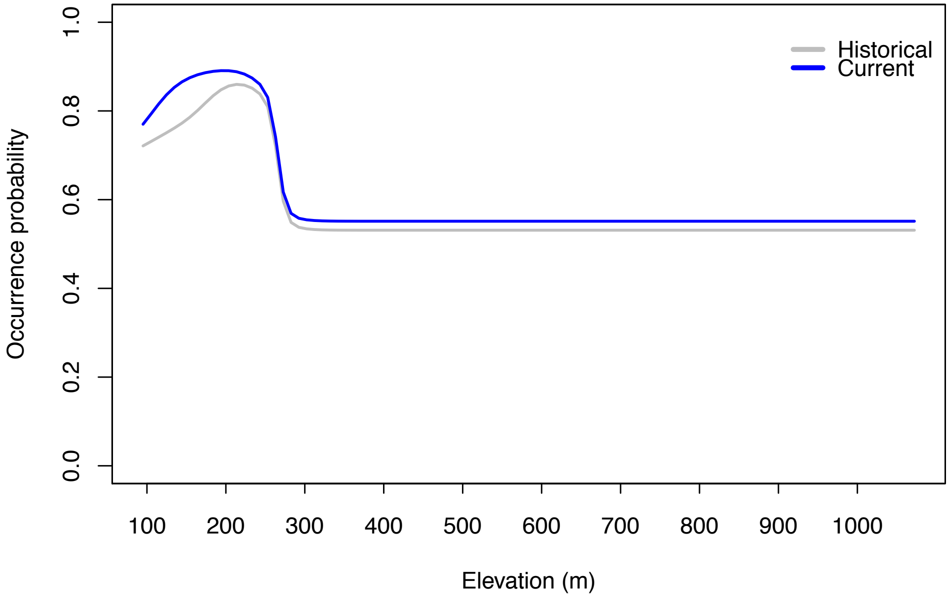

Occupancy models	Cumulative AIC weight
Constant	0.21
**Elev**	**0.31**
Elev+Elev^2	0.11
Era	0.09
Era+Elev	0.14
Era+Elev+Elev^2	0.05
Era*Elev	0.08
Era*(Elev+Elev^2)	0.01

Optimal historical= 213 m
Optimal modern= 193 m

M

13 – Icterus portoricensis

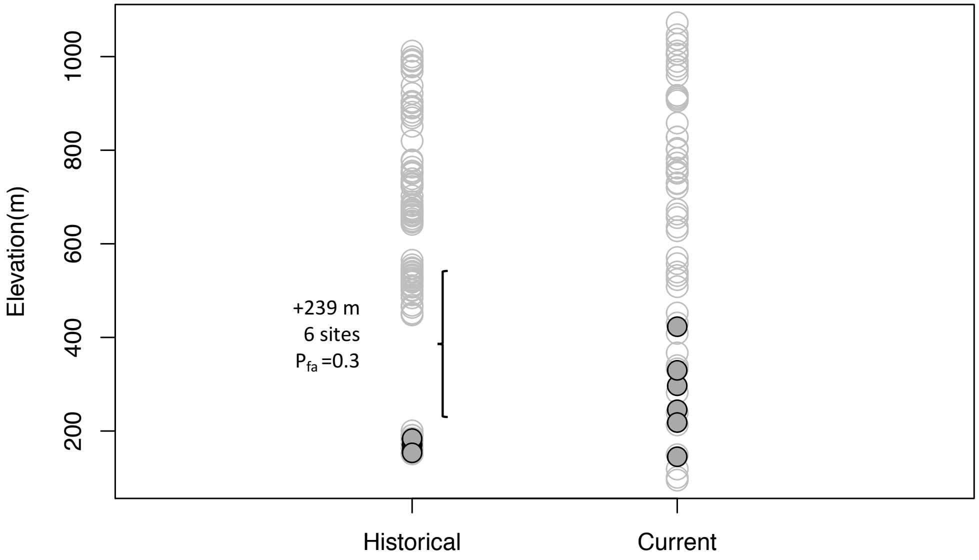

**Occupancy models**	**Cumulative AIC weight**
Constant	0.09
Elev	0.24
Elev+Elev^2	0.09
Era	0.04
**Era+Elev**	**0.25**
Era+Elev+Elev^2	0.09
Era*Elev	0.14
Era*(Elev+Elev^2)	0.05

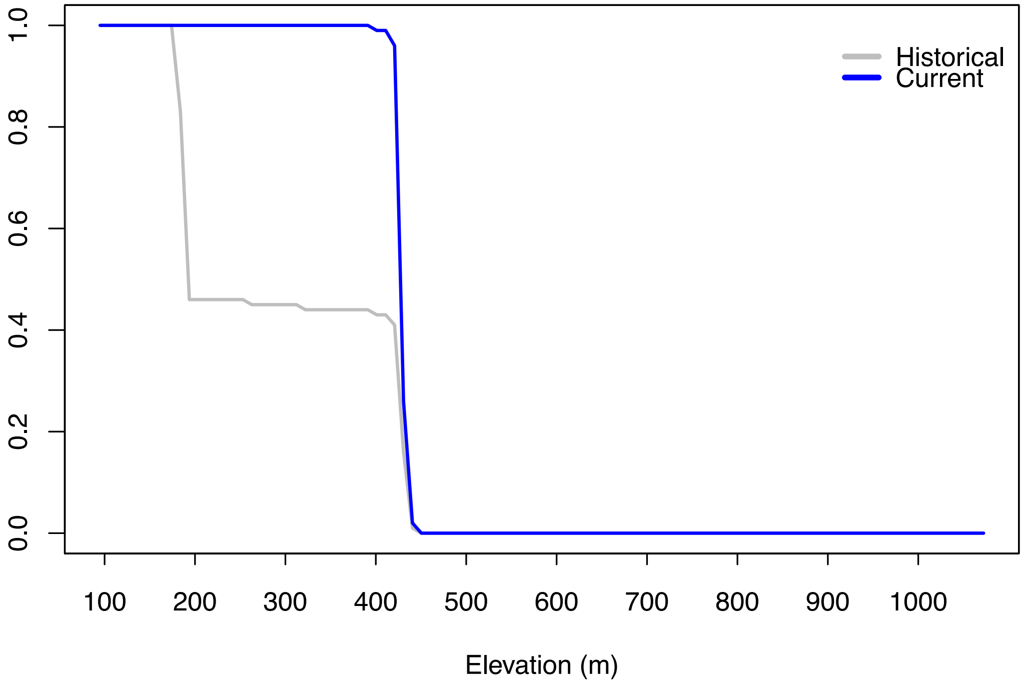

Optimal historical= 95-174 m
Optimal modern= 95 - 391 m

N

14 – *Margarops fuscatus*

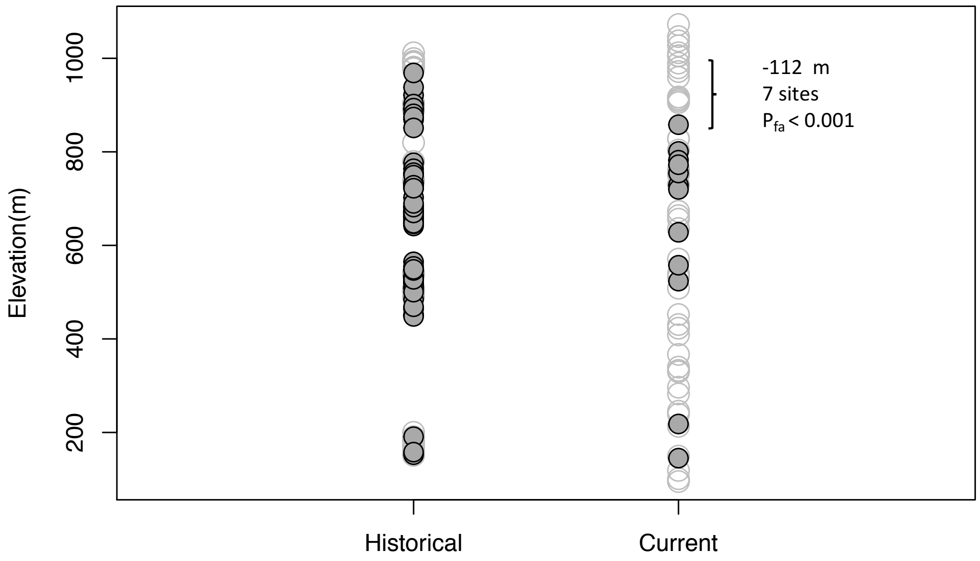

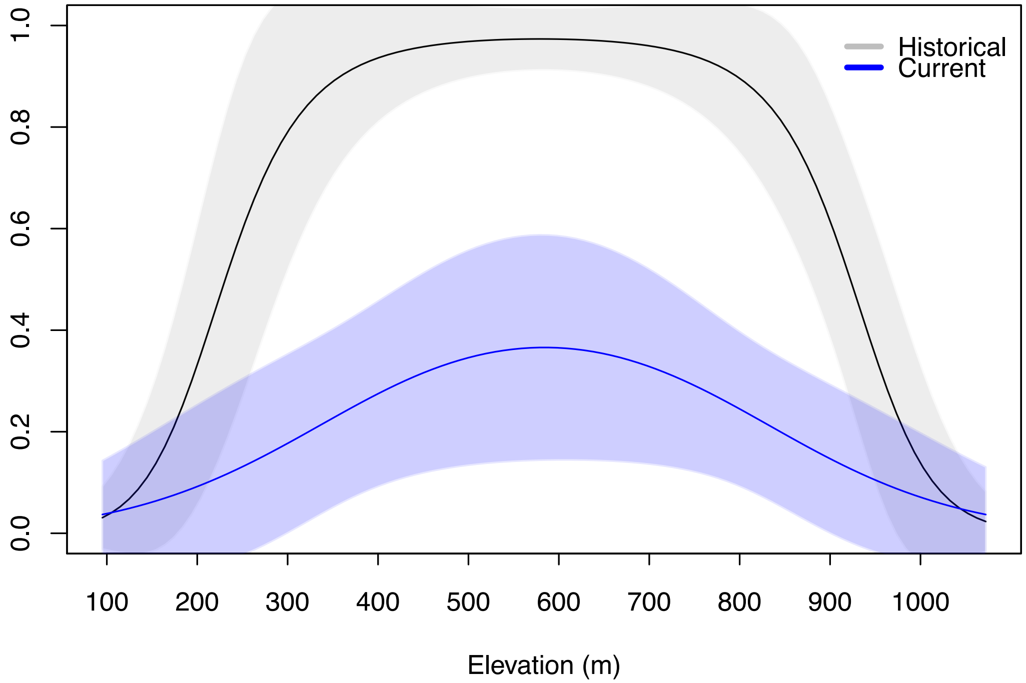

**Occupancy models**	**Cumulative AIC weight**
Constant	0.00
Elev	0.00
Elev+Elev^2	0.00
Era	0.05
Era+Elev	0.13
Era+Elev+Elev^2	0.28
Era*Elev	0.00
**Era*(Elev+Elev^2)**	**0.54**

Optimal historical= 480 - 677 m
Optimal modern= 568 - 598 m

O

15 – Zenaida aurita

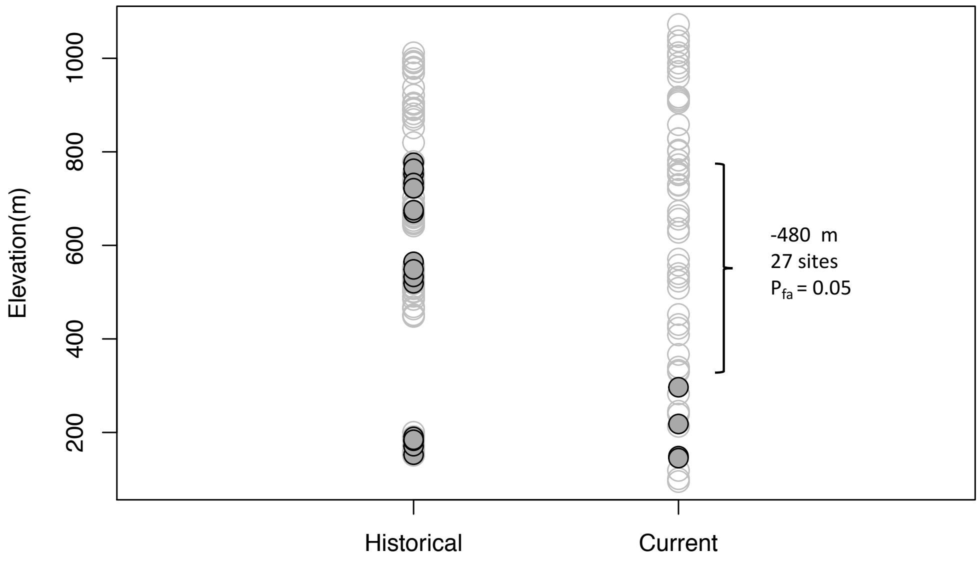

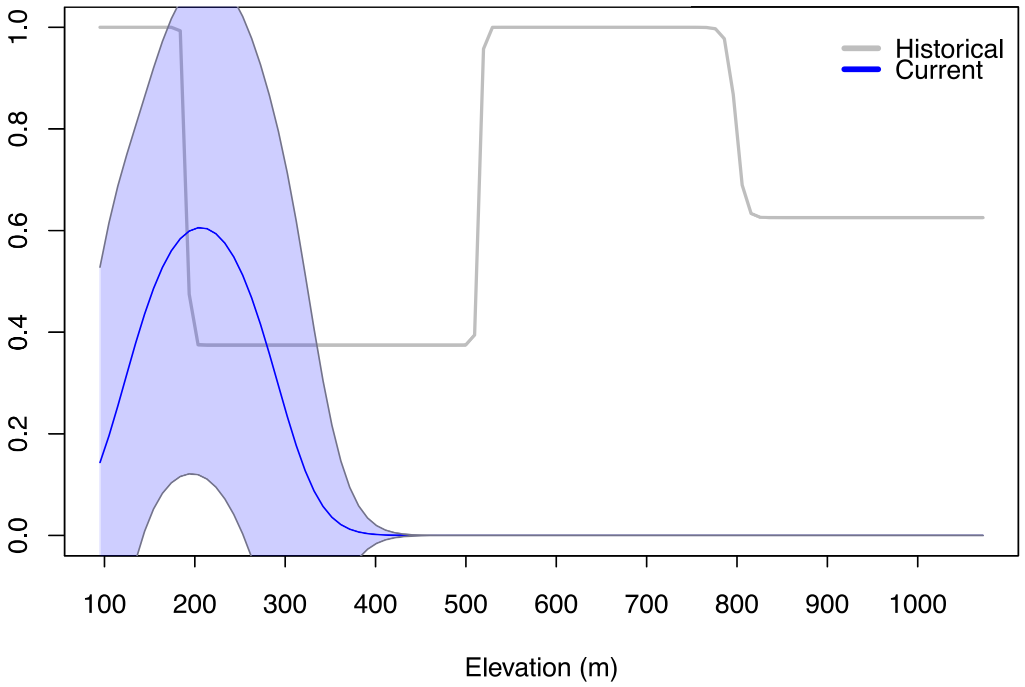

**Occupancy models**	**Cumulative AIC weight**
Constant	0.00
Elev	0.00
Elev+Elev^2	0.00
Era	0.00
Era+Elev	0.01
Era+Elev+Elev^2	0.33
Era*Elev	0.00
**Era*(Elev+Elev^2)**	**0.65**

Optimal historical= 95-776 m
Optimal modern= 203 m

P

16 – Loxigilla portoricensis

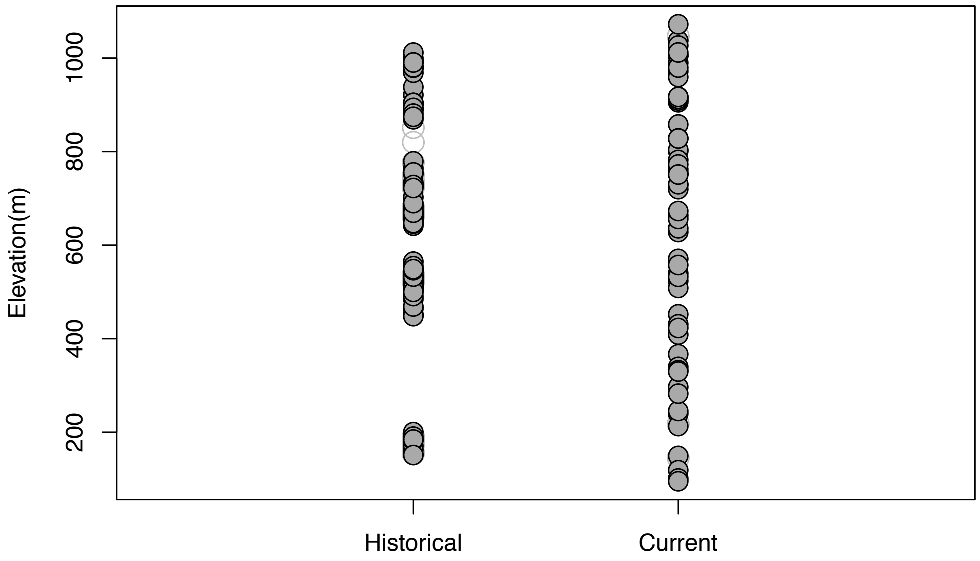

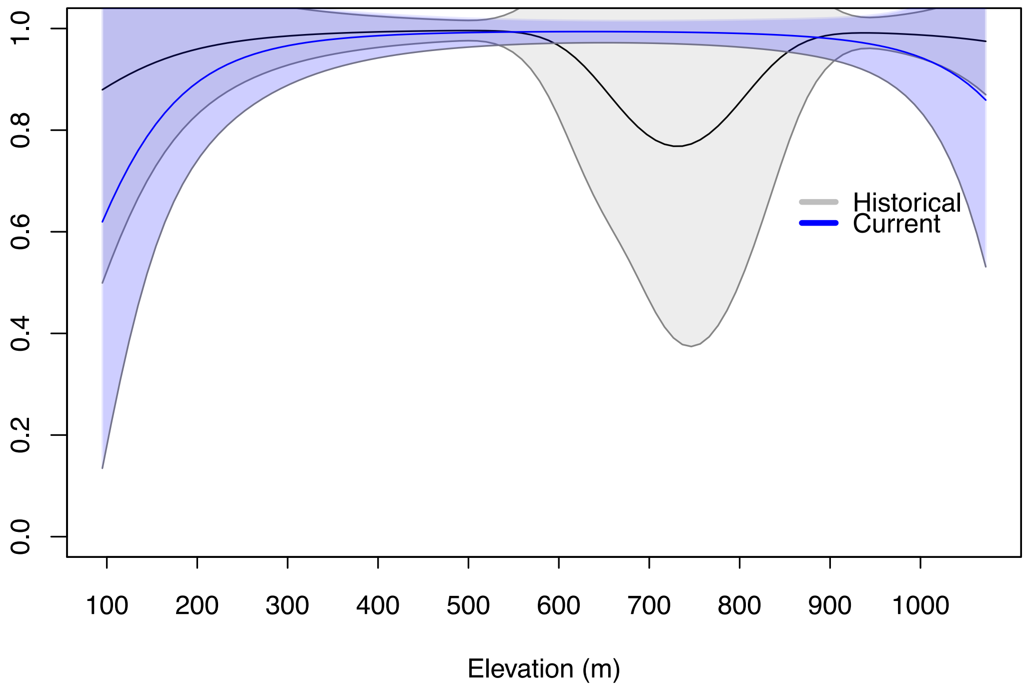

Occupancy models	Cumulative AIC weight
Constant	0.12
Elev	0.13
Elev+Elev^2	0.17
Era	0.05
Era+Elev	0.07
Era+Elev+Elev^2	0.06
Era*Elev	0.03
**Era*(Elev+Elev^2)**	**0.37**

Optimal historical= 450 -529 m
Optimal current= 401 - 855m

Q

17 – Todus mexicanus

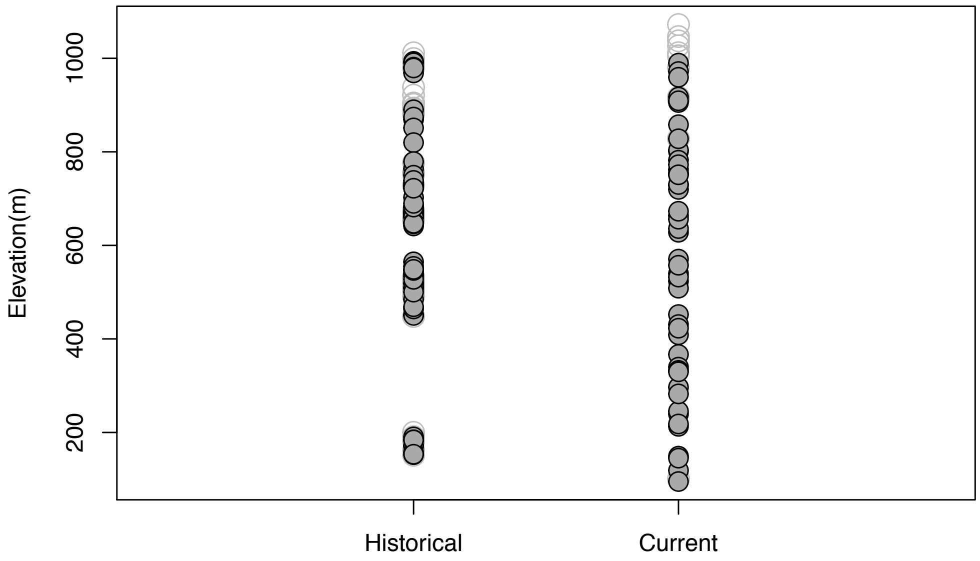

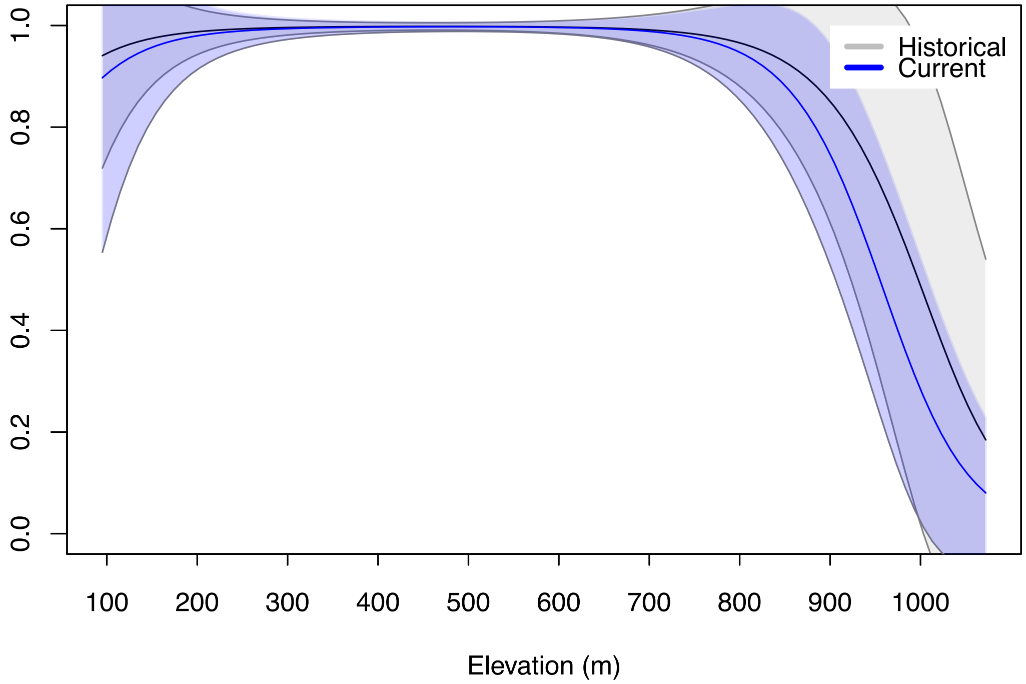

Occupancy models	Cumulative AIC weight
Constant	0.00
Elev	0.22
Elev+Elev^2	0.22
Era	0.00
Era+Elev	0.21
**Era+Elev+Elev^2**	**0.23**
Era*Elev	0.09
Era*(Elev+Elev^2)	0.04

Optimal historical= 282-647 m
Optimal modern= 331- 628 m

R

18 – Patagioenas squamosa

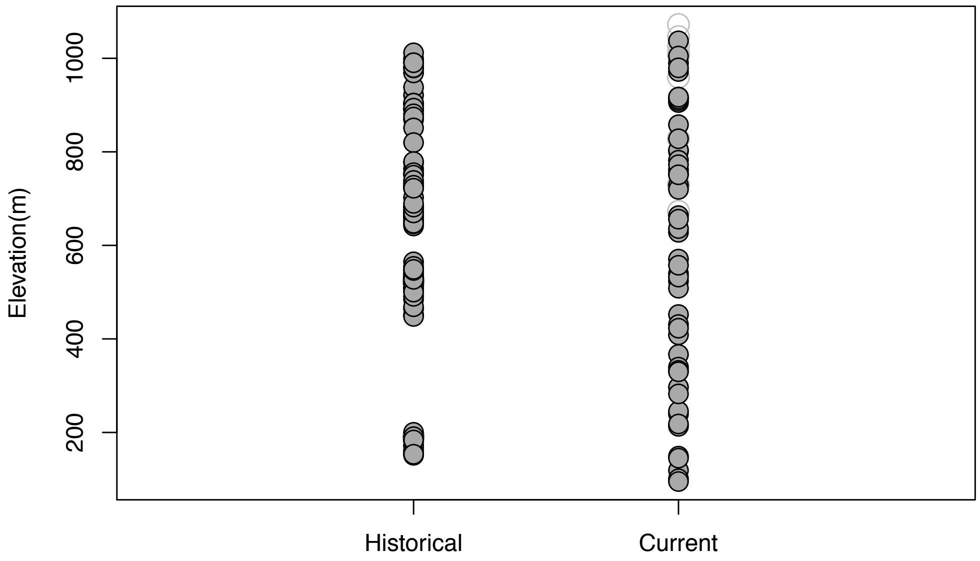

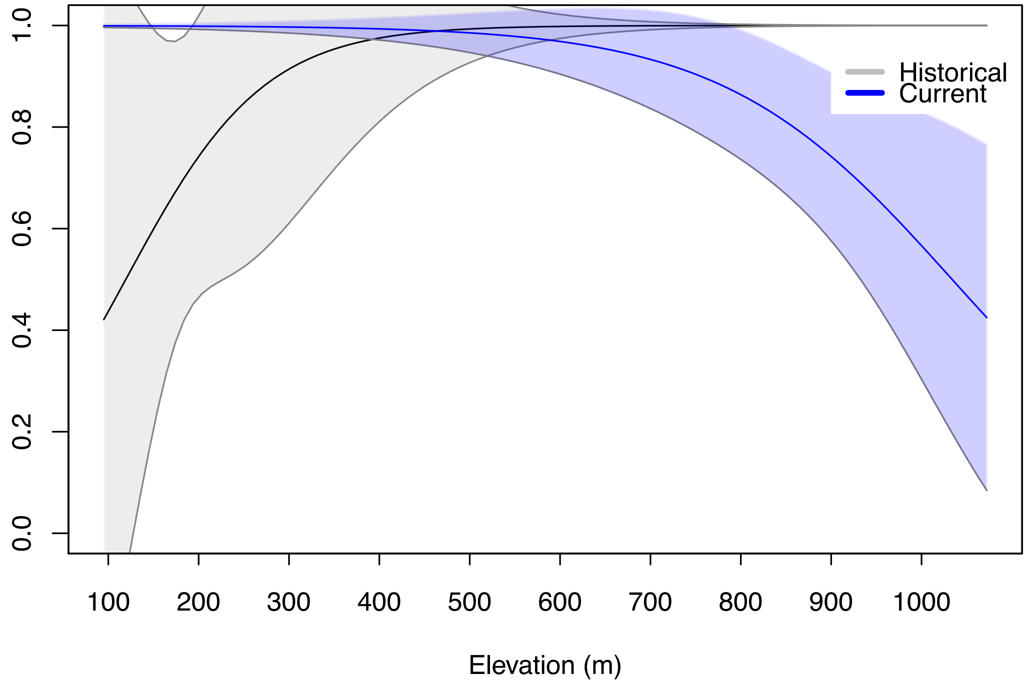

Occupancy models	Cumulative AIC weight
Constant	0.00
Elev	0.00
Elev+Elev^2	0.00
Era	0.00
Era+Elev	0.04
Era+Elev+Elev^2	0.01
**Era*Elev**	**0.83**
Era*(Elev+Elev^2)	0.12

Optimal historical= 529-1072 m
Optimal modern= 95-371 m

S

19 – Turdus plumbeus

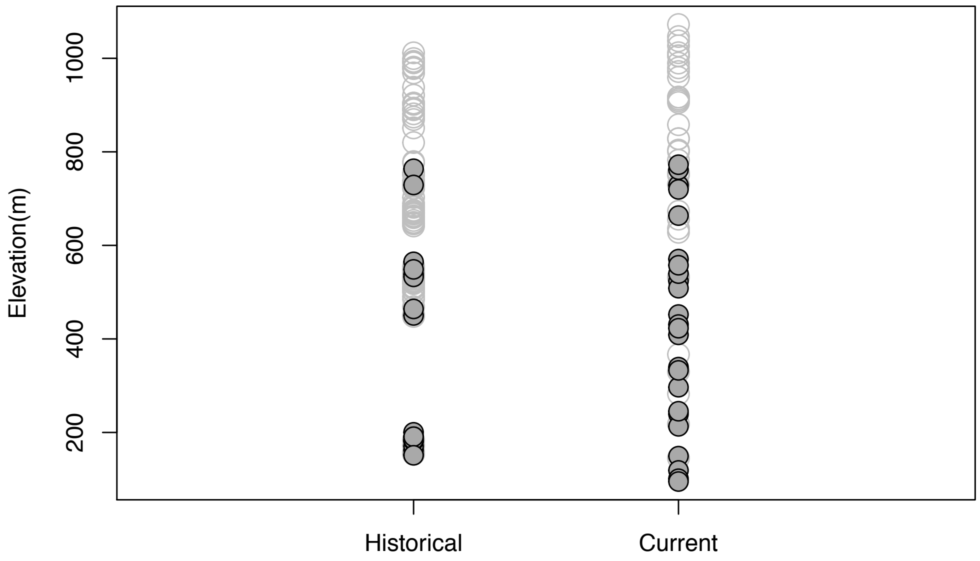

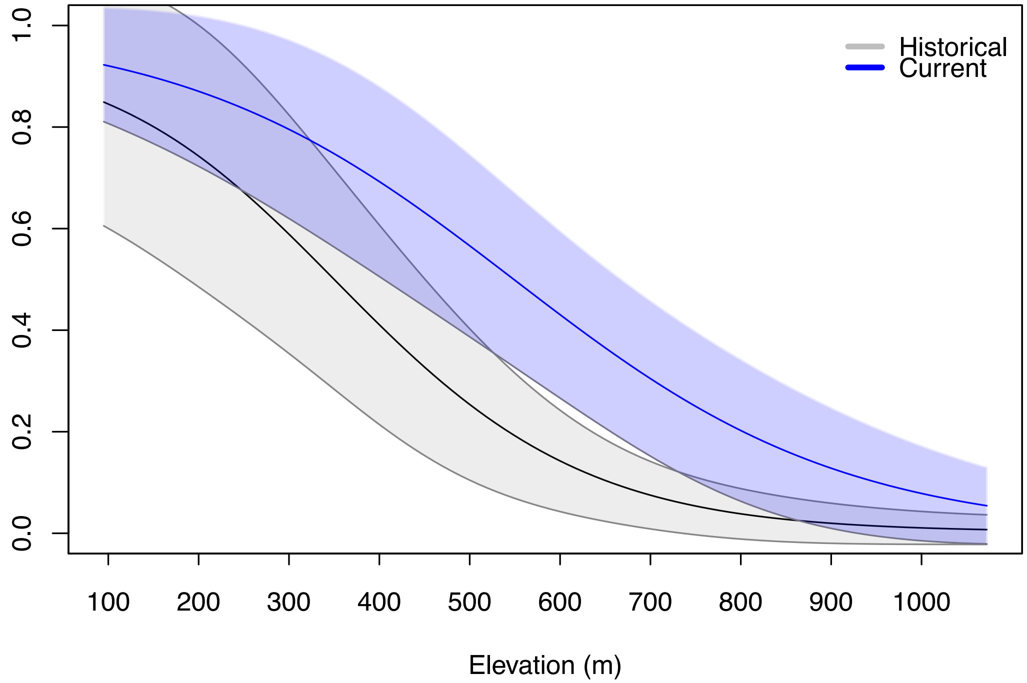

**Occupancy models**	**Cumulative AIC weight**
Constant	0.00
Elev	0.01
Elev+Elev^2	0.00
Era	0.00
Era+Elev	0.28
Era+Elev+Elev^2	0.19
Era*Elev	0.19
**Era*(Elev+Elev^2)**	**0.34**

Optimal historical= 95 m
Optimal modern= 95 m

T

20 – Tyrannus dominicensis

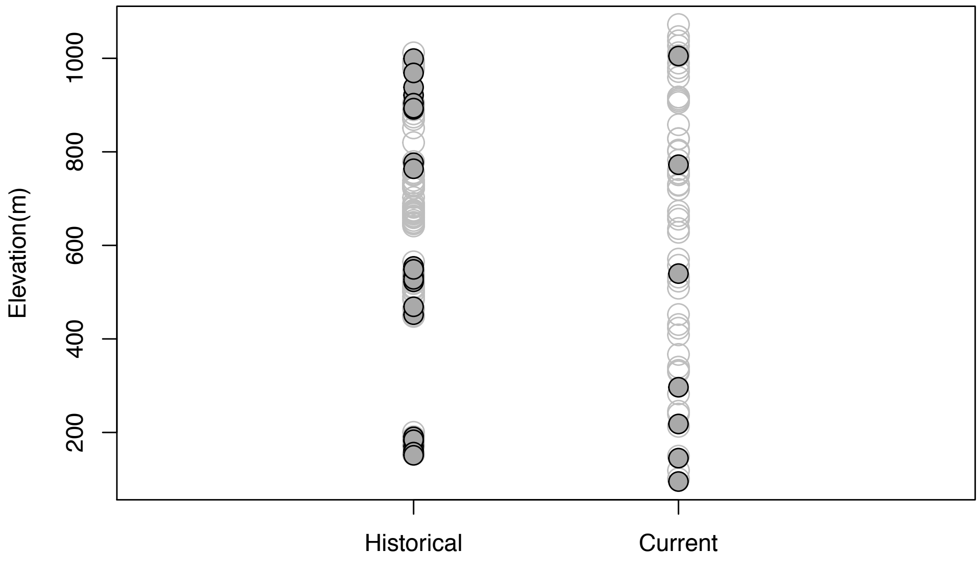

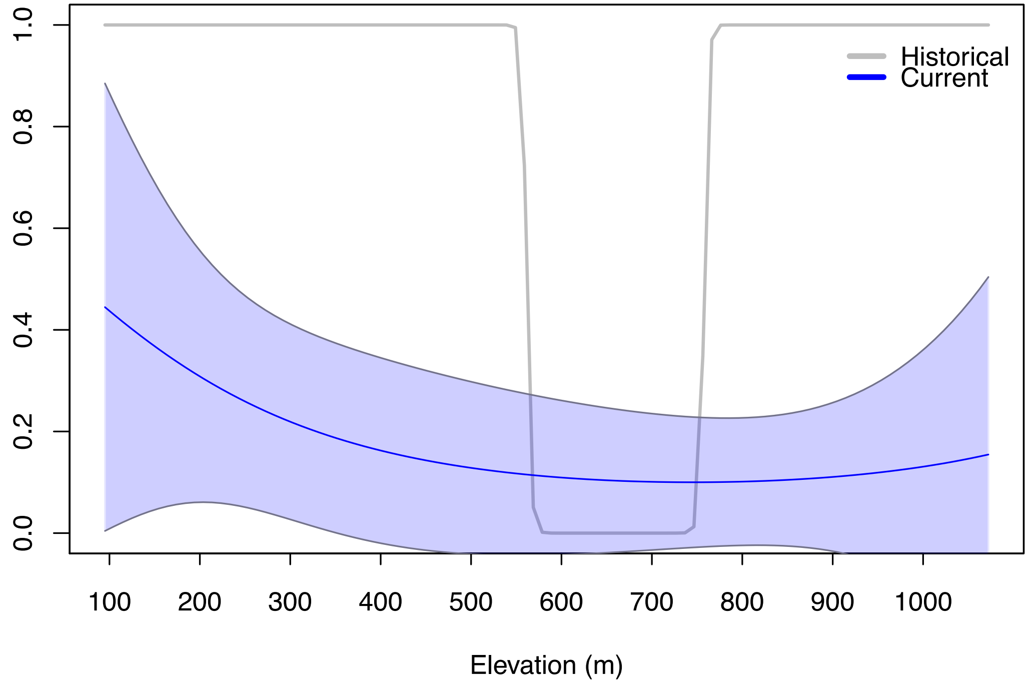

**Occupancy models**	**Cumulative AIC weight**
Constant	0.00
Elev	0.00
Elev+Elev^2	0.00
Era	0.01
Era+Elev	0.01
Era+Elev+Elev^2	0.01
Era*Elev	0.00
**Era*(Elev+Elev^2)**	**0.98**

Optimal historical= 95 - 1072 m
Optimal modern= 95 m

U

21 – Coereba flaveola

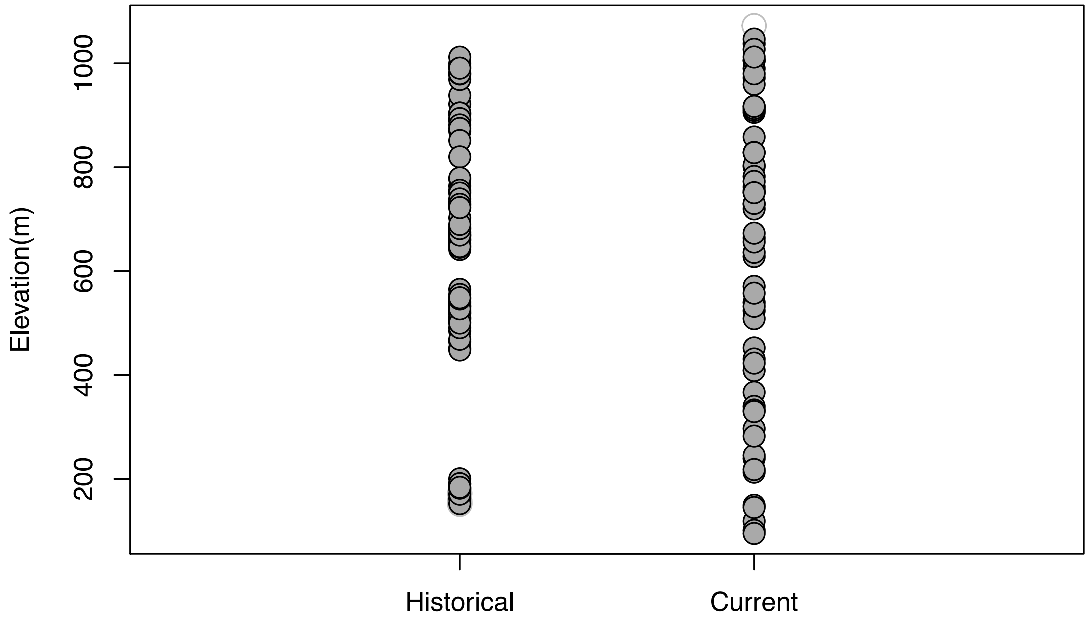

Species not analyzed with occupancy models – Detections in all but one site
