# Supplementary material for: Have bird distributions shifted along an elevational gradient on a tropical mountain?
Source: Ecol Evol. 2017 Oct 20;7(23):9914–24. doi: 10.1002/ece3.3520 (PMC5723601; doi:10.1002/ece3.3520)
Supplement: Supplementary file 2 [file ECE3-7-9914-s002.docx]

# APPENDIX

Table S1: Candidate model sets for the occupancy analysis.

| **Model** | **Occupancy** | **Detection** |
| --- | --- | --- |
| 1 | (.) | (.) |
| 2 | Era | (.) |
| 3 | Elevation | (.) |
| 4 | Elevation+Elevation^2^ | (.) |
| 5 | Era+Elevation | (.) |
| 6 | Era+Elevation+Elevation^2^ | (.) |
| 7 | Era*Elevation | (.) |
| 8 | Era*(Elevation+Elevation^2^) | (.) |
| 9 | (.) | Era |
| 10 | Era | Era |
| 11 | Elevation | Era |
| 12 | Elevation+Elevation^2^ | Era |
| 13 | Era+Elevation | Era |
| 14 | Era+Elevation+Elevation^2^ | Era |
| 15 | Era*Elevation | Era |
| 16 | Era*(Elevation+Elevation^2^) | Era |
| 17 | (.) | Elevation |
| 18 | Era | Elevation |
| 19 | Elevation | Elevation |
| 20 | Elevation+Elevation^2^ | Elevation |
| 21 | Era+Elevation | Elevation |
| 22 | Era+Elevation+Elevation^2^ | Elevation |
| 23 | Era*Elevation | Elevation |
| 24 | Era*(Elevation+Elevation^2^) | Elevation |
| 25 | (.) | Elevation+Elevation^2^ |
| 26 | Era | Elevation+Elevation^2^ |
| 27 | Elevation | Elevation+Elevation^2^ |
| 28 | Elevation+Elevation^2^ | Elevation+Elevation^2^ |
| 29 | Era+Elevation | Elevation+Elevation^2^ |
| 30 | Era+Elevation+Elevation^2^ | Elevation+Elevation^2^ |
| 31 | Era*Elevation | Elevation+Elevation^2^ |
| 32 | Era*(Elevation+Elevation^2^) | Elevation+Elevation^2^ |

Table S2: Species list with number of detections and occupied sites for each time period.

| **Species** | **Detections(H)** | **Sites(H)** | **Detections(C)** | **Sites(C)** |
| --- | --- | --- | --- | --- |
| *Accipiter striatus venator* | 0 | 0 | 1 | 1 |
| *Amazona vittata* | 11 | 9 | 1 | 1 |
| *Anthracothorax dominicus* | 1 | 1 | 0 | 0 |
| *Anthracothorax viridis* | 1 | 1 | 1 | 1 |
| *Buteo jamaicensis* | 4 | 4 | 7 | 5 |
| *Buteo platypterus brunnescens* | 1 | 1 | 1 | 1 |
| *Chlorostilbon maugeaus* | 10 | 9 | 8 | 4 |
| *Coccyzus minor* | 2 | 2 | 7 | 5 |
| *Coccyzus vieilloti* | 38 | 29 | 95 | 26 |
| *Coereba flaveola* | 265 | 101 | 374 | 59 |
| *Columbina passerina* | 7 | 6 | 0 | 0 |
| *Contopus latirostris* | 0 | 0 | 1 | 1 |
| *Euphonia musica* | 5 | 5 | 13 | 9 |
| *Geotrygon montana* | 8 | 7 | 46 | 18 |
| *Geotrygon mystacea* | 1 | 1 | 0 | 0 |
| *Icterus portoricensis* | 5 | 5 | 7 | 6 |
| *Loxigilla portoricensis* | 133 | 79 | 322 | 57 |
| *Margarops fuscatus* | 152 | 71 | 56 | 12 |
| *Megascops nudipes* | 7 | 6 | 58 | 20 |
| *Melanerpes portoricensis* | 72 | 57 | 128 | 35 |
| *Molothrus bonariensis* | 0 | 0 | 2 | 1 |
| *Mniotilta varia* | 7 | 7 | 0 | 0 |
| *Myiarchus antillarum* | 25 | 19 | 11 | 8 |
| *Nesospingus speculiferus* | 73 | 52 | 239 | 51 |
| *Parkesia motacilla* | 2 | 2 | 0 | 0 |
| *Patagioenas leucocephala* | 0 | 0 | 2 | 1 |
| *Patagioenas squamosa* | 244 | 103 | 260 | 51 |
| *Setophaga adelaide* | 6 | 6 | 2 | 1 |
| *Setophaga americana* | 1 | 1 | 27 | 9 |
| *Setophaga angelae* | 6 | 6 | 113 | 22 |
| *Setophaga caerulescens* | 1 | 1 | 0 | 0 |
| *Setophaga ruticilla* | 8 | 7 | 25 | 6 |
| *Spindalis portoricensis* | 59 | 62 | 312 | 57 |
| *Tiaris bicolor* | 43 | 31 | 0 | 0 |
| *Tiaris olivaceus* | 2 | 2 | 0 | 0 |
| *Todus mexicanus* | 89 | 65 | 215 | 48 |
| *Turdus plumbeus* | 34 | 20 | 130 | 24 |
| *Tyrannus dominicensis* | 35 | 28 | 21 | 7 |
| *Vireo altiloquus* | 72 | 39 | 189 | 31 |
| *Vireo latimeri* | 1 | 1 | 0 | 0 |
| *Zenaida asiatica* | 0 | 0 | 22 | 8 |
| *Zenaida aurita* | 25 | 19 | 11 | 4 |

Table S3: Summary of studies that have tested for shifts in elevational distribution of birds.

| **Study** | **Date** | **Elevation** | **Temperature** | **Total** | **Upslope** | **Downslope** | **No change** |
| --- | --- | --- | --- | --- | --- | --- | --- |
| Harris *et al.*, 2012 | 1998–2006 | 1450-4095 | 0.48ºC (mean annual) | 58 | 24 | 14 | - |
| Forero-Medina *et al.*, 2011 | 1969–2010 | 690-2220 | 0.019ºC per year | 55 | 36 | 12 | 7 |
| Peh, 2007 | 1975–2000 | 0-5881 (?) | 0.3ºC (1987-2007?) | 485 | 129/30 | 66/79 | 264 |
| Freeman and Freeman, 2014 | 1965­–2012 | 1130-2520/800-1600 | 0.39ºC and 0.46ºC (annual mean) | 131 | 104/39 | 41/14 |  |
| Tingley *et al.*, 2012 | 1911–2009 | 80–2751/65–3226/61–3356 | 3.7ºC (average minimum monthly) | 99 | 53% | 46% | >50% in each region |
| Popy, Bordignon and Prodon, 2010 | 1992–2005 | 550-2556 | 1.0 ºC (mean) /1.4 ºC (maximum) | 61 | 42* | 19 | - |
| Archaux, 2004 | 1970–2000 | 620-3099/ 350-1912 | 1.3 ° C/ 2.3 ° C (mean annual) | 24/17 | 4 | 6 | - |
